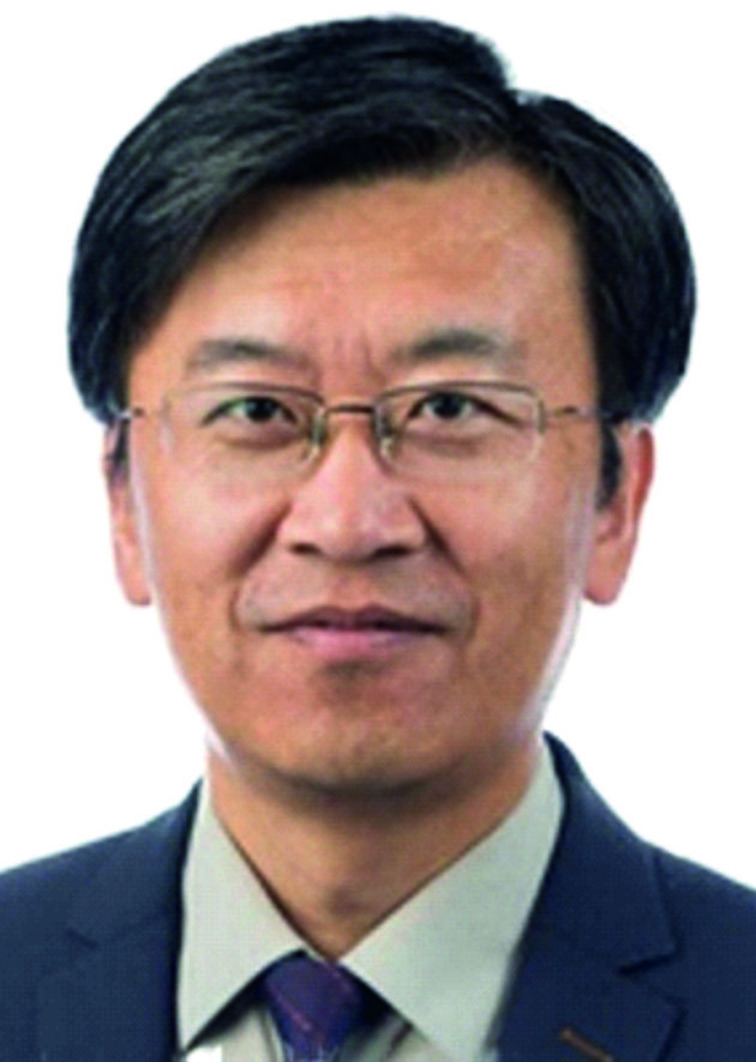# Advances in Two‐Electron Water Oxidation Reaction for Hydrogen Peroxide Production: Catalyst Design and Interface Engineering

**DOI:** 10.1002/cssc.202401100

**Published:** 2024-10-23

**Authors:** Huixuan Cao, Ge Chen, Yong Yan, Dong Wang

**Affiliations:** ^1^ Beijing Key Laboratory for Green Catalysis and Separation Department of Chemical Engineering and Technology College of Materials and Manufacturing Beijing University of Technology Beijing 100124 P. R. China; ^2^ Center of Excellence for Environmental Safety and Biological Effects Beijing Key Laboratory for Green Catalysis and Separation Department of Chemistry College of Chemistry and Life Science Beijing University of Technology Beijing 100124 P. R. China; ^3^ Fachgebiet Werkstoffe der Elektrotechnik Institute of Materials Science & Engineering and Institute of Micro- and Nanotechnologies MarcoNano® TU Ilmenau Gustav-Kirchhoff-Str. 5 98693 Ilmenau Germany

**Keywords:** Electrocatalyst, Electrolyte, 2e-WOR, H_2_O_2_, Interface

## Abstract

Hydrogen peroxide (H_2_O_2_) is a versatile and zero‐emission material that is widely used in the industrial, domestic, and healthcare sectors. It is clear that it plays a critical role in advancing environmental sustainability, acting as a green energy source, and protecting human health. Conventional production techniques focused on anthraquinone oxidation, however, electrocatalytic synthesis has arisen as a means of utilizing renewable energy sources in conjunction with available resources like oxygen and water. These strides represent a substantial change toward more environmentally and energy‐friendly H_2_O_2_ manufacturing techniques that are in line with current environmental and energy goals. This work reviews recent advances in two‐electron water oxidation reaction (2e‐WOR) electrocatalysts, including design principles and reaction mechanisms, examines catalyst design alternatives and experimental characterization techniques, proposes standardized assessment criteria, investigates the impact of the interfacial milieu on the reaction, and discusses the value of in situ characterization and molecular dynamics simulations as a supplement to traditional experimental techniques and theoretical simulations. The review also emphasizes the importance of device design, interface, and surface engineering in improving the production of H_2_O_2_. Through adjustments to the chemical microenvironment, catalysts can demonstrate improved performance, opening the door for commercial applications that are scalable through tandem cell development.

## Graphical abstract

This paper reviews recent advances in two‐electron water oxidation reaction electrocatalysts for H_2_O_2_ production, including mechanistic studies, catalyst design strategies, and the impact of Three‐Phase Interface Engineering. It combines theoretical modeling and experimental studies to improve H_2_O_2_ production.

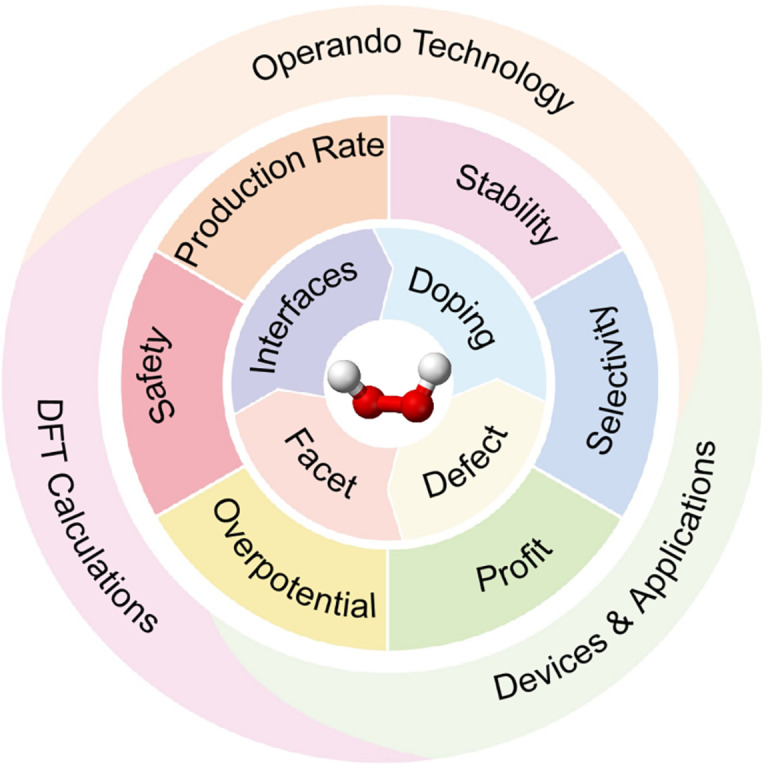

## 1 Introduction

Hydrogen peroxide (H_2_O_2_) is a zero‐emission and highly effective chemical oxidant, with applications ranging from industry to household usage,^[10]^ such as paper bleaching,^[12]^ wastewater treatment^[14]^ and sterilization,^[18]^ encompassing the deadly Corona Virus Disease in 2019 (COVID‐19), which has a positive impact on the environment and is crucial for human health. High‐purity H_2_O_2_ is also used for semiconductor cleaning agents, etching machines, and photoresist removers. In the advanced oxidation (AO) process, H_2_O_2_ efficiently degrades organic pollutants by generating numerous reactive oxygen species (ROS).^[21]^ In addition to these well‐known applications, H_2_O_2_ has been developed as a green energy carrier and self‐propelled, which releases 96 kJ mol^−1^ of energy through exothermic chemical decomposition. This controlled H_2_O_2_ breakdown enables the conversion of chemical energy into kinetic energy in a variety of contexts, such as rocket propellant and machine fuel.^[22]^ Furthermore, H_2_O_2_ storage does not require pressurization or low temperatures, avoiding many issues associated with industrial storage; nonetheless, it should be careful to minimize decomposition by avoiding combining metal ion contaminants, exposure to ultraviolet (UV) light, and excessive heat.

The conventional method for the production of H_2_O_2_ is through the process of anthraquinone oxidation (AO).^[23]^ Standard AO processes encompass a variety of steps, such as catalytic hydrogenation, oxidation reactions, extraction, distillation, concentration, and purification.^[24]^ Nonetheless, utilizing industrial AO incurs evident disadvantages such as high overhead costs, explosion hazards, and transportation issues.[^23^, ^25^] The direct synthesis of H_2_O_2_ from gaseous mixtures of H_2_ and O_2_ at high pressures is relatively straightforward when noble metals such as Pd are used as catalysts.^[26]^ However, the generation of H_2_O_2_ faces the challenge of competing with the thermodynamically favorable H_2_ combustion reaction. Besides the risk of H_2_ explosion, the inactive catalysts as well as the low selectivity of H_2_O_2_ hinder the technology′s advancement.[^19^, ^40^] On‐site (photo)electrochemical systems can function at room temperature and atmospheric pressure with a minimal capital investment. Besides, the electrical energy involved in the electrochemical process can be converted from several renewable energy sources, including solar, wind and tidal energy. Moreover, no organic chemicals are needed in the (photo)electrochemical process, suggesting an eco‐friendly approach. Significant progress has been made in the generation of H_2_O₂ production during the past several years. The finding of novel catalysts, mechanic understanding, and the design of H₂O₂ (photo)electrochemical devices are some of the research advances.^[41]^

**Figure 1:**
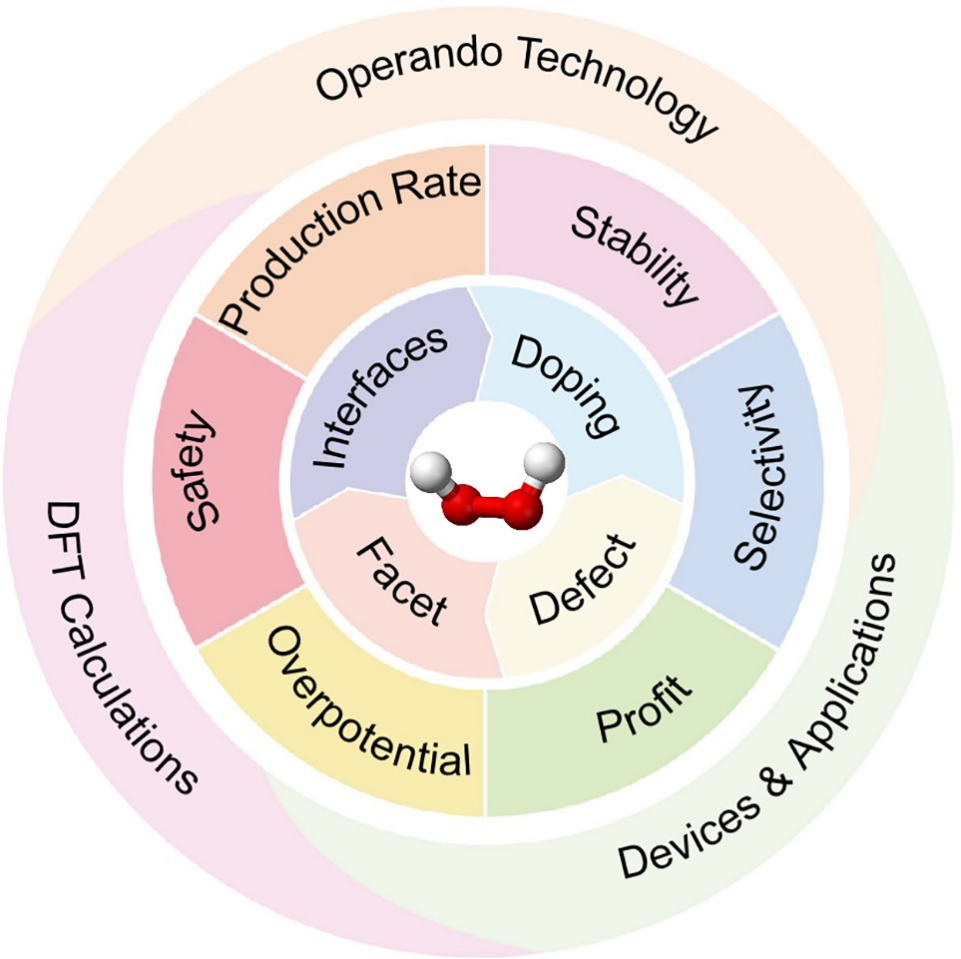
Review of progress in 2e‐WOR.

**Figure 2:**
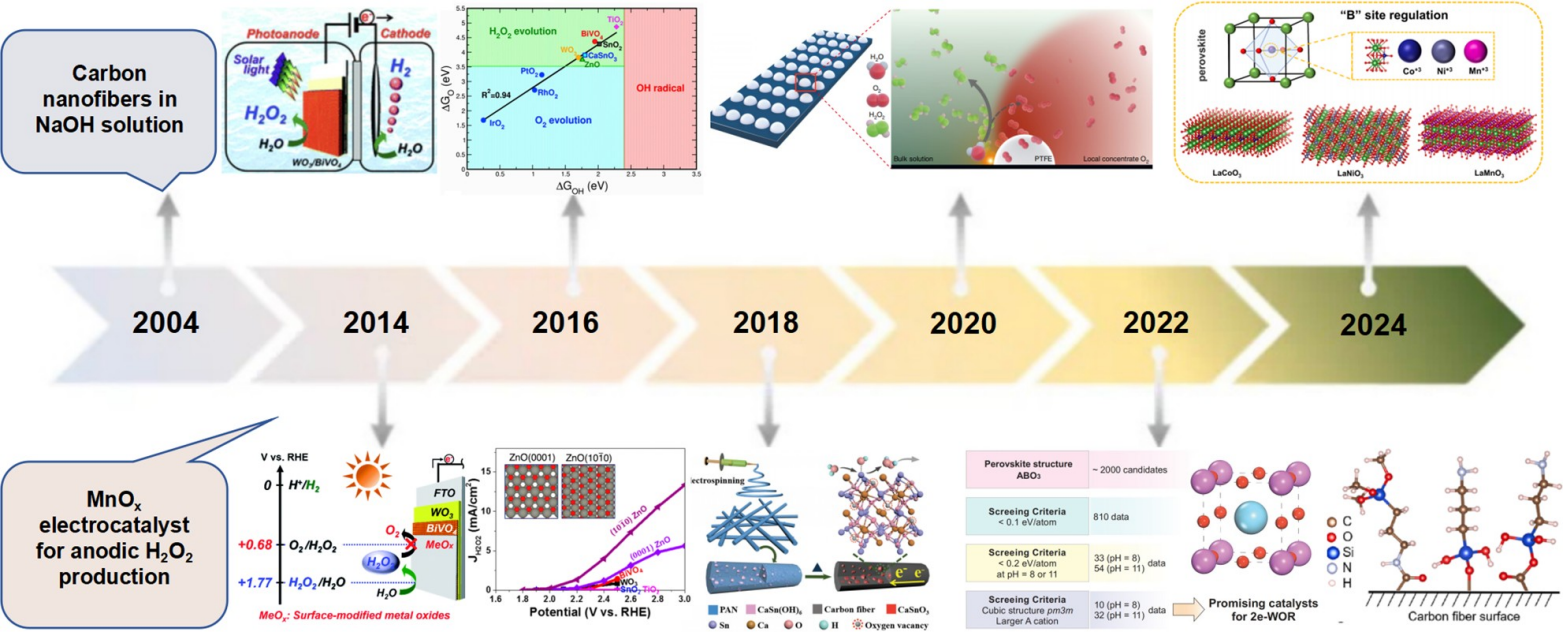
Timeline of previous studies on the 2e‐WOR for anodic H_2_O_2_ production. Reprinted (adapted) with permission from ref. [1] Copyright 2016 Royal Society of Chemistry. Reprinted (adapted) with permission from ref. [2] Copyright 2017 Royal Society of Chemistry. Reprinted (adapted) with permission from ref. [3] Copyright 2020 American Chemical Society. Reprinted (adapted) with permission from ref. [4] Copyright 2019 American Chemical Society. Reprinted (adapted) with permission from ref. [5] Copyright 2021 Wiley‐VCH. Reprinted (adapted) with permission from ref. [6] Copyright 2020 Nature Publishing Group. Reprinted (adapted) with permission from ref. [7] Copyright 2022 Springer Nature. Reprinted (adapted) with permission from ref. [8] Copyright 2023 Elsevier. Reprinted (adapted) with permission from ref. [9] Copyright 2024 Elsevier.

In recent years, utilizing water (H_2_O) and molecular oxygen (O_2_) as the green raw materials, electricity and sunlight as the energy source can reach energy transformation and H_2_O_2_ storage.[^34^, ^42^] Electrocatalytic production of H_2_O_2_ is currently predominantly achieved through the cathodic oxygen reduction reaction (2e‐ORR)^[45]^ and the anodic water oxidation reaction (2e‐WOR).[^1^, ^2^, ^4^, ^54^, ^59^] The 2e‐ORR process is considered harmful for fuel cells, but provides an opportunity for hydrogen peroxide synthesis. The process involves the requirement of gas diffusion electrode and continuous O_2_ purging, which leads to a more complicated operation.^[61]^ Unlike the 2e‐ORR, the 2e‐WOR does not rely on continuous O_2_ purging, providing a new approach for electrochemical H_2_O_2_ production. The process is simple and has more practical application prospects.^[63]^ Since an enormous body of work has been published about the development of 2e‐WOR for H_2_O_2_ production, we have reviewed the recent advances in this field, as shown in Figure 1. We first discuss the theoretical aspects of the rational design of 2e‐WOR electrocatalysts, including different possible mechanisms, analysing the free energy diagram, and understanding trends of activity among different catalyst materials.^[66]^ Meanwhile, we discuss the effects of solvation and the energetics of reaction intermediates, regarding the application and necessity of MD simulations to the exploration of liquid electrolytes in interfacial structures. Together, these theoretical frameworks provide important insights and guidance on the selection of 2e‐WOR catalysts for experimental work. In experimental methods, we discuss the H_2_O_2_ quantification methods and characterizing techniques for 2e‐WOR electrocatalysts, including in‐situ spectroscopy and isotope‐labeled mass spectrometry to further identify the adsorption of intermediates or the dynamic evolution of active sites during the reaction. We summarize the most recent performance benchmarks of previously studied electrocatalysts, as shown in Figure 2. Several standardized recommendations were proposed for the assessment of electrocatalyst performance metrics in this review. We provide a concise overview of the recent catalyst design, with a specific emphasis on the surface, interface, and device engineering in the production of H_2_O_2_. By manipulating the chemical microenvironment that governs the interaction between catalysts, reactants, intermediates, products, and electrolytes in H_2_O_2_ production, the existing active sites can be modified to achieve better catalytic performance. Considering complementary to the catalyst design, the ‘device engineering’ part is summarized for understanding a maximum H_2_O_2_ output, and thus advancing laboratory‐scale production to larger‐scale industrial production.

## 2 Electrochemical 2 e‐WOR Mechanistic Studies

### 2.1 Thermodynamics of 2 e‐WOR

The process of water oxidation is linked to three distinct pathways.^[66]^ Three mechanisms are distinguished based on the quantity of electron transfer. Using density functional theory (DFT), Viswanathan et al. demonstrated that the selectivity between 2e‐ and 4e‐WOR can be explained by the adsorption free energy of OH*. In 2017, Siahrostami et al. discussed issues related to 1e‐WOR, which generates OH radicals, and the subsequent water oxidation to H_2_O_2_.^[68]^ They discovered that thermodynamic barriers restrain the creation of an active and selective catalyst for this process.

Equations (1–3) describe the reaction processes for the three WORs.^[20]^ The initial step shared by all three WORs is the formation of adsorbed OH (OH*). The hydroxyl radical (OH⋅) is produced via the 1e‐WOR pathway and is a highly attractive method for degrading pollutants and disinfecting water (as shown in Equation 3). Nonetheless, the reaction′s progress is greatly limited by the extremely short lifespan of OH⋅ and the highest equilibrium potential (E°=2.38 V vs. RHE) of 1e‐WOR.

The 4e‐WOR:

(1) $$2H_{2}O\to O_{2}+4\left(H^{+}+e^{-}\right)\;\;\left(E^{\circ }=1.23V_{RHE}\right)$$

(1a) $$*+H_{2}O\to OH*+\left(H^{+}+e^{-}\right)$$

(1b) $$OH*\to O*+\left(H^{+}+e^{-}\right)$$

(1c) $$O*+H_{2}O\to OOH*+\left(H^{+}+e^{-}\right)$$

(1d) $$OOH*\to *+O_{2}+\left(H^{+}+e^{-}\right)$$

The 2e‐WOR:

(2) $$2H_{2}O\to H_{2}O_{2}+2\left(H^{+}+e^{-}\right)\;\;\left(E^{\circ }=1.76V_{RHE}\right)$$

(2a) $$*+H_{2}O\to OH*+\left(H^{+}+e^{-}\right)\;\mathrm{the}\;\mathrm{same}\;\mathrm{as}\;\mathrm{Equation}\;1A$$

(2b) $$OH*+H_{2}O\to H_{2}O_{2}+\left(H^{+}+e^{-}\right)+*$$

The one‐electron WOR (1e‐WOR):

(3) $$H_{2}O\to OH{*}_{\left(\mathrm{aq}\right)}+\left(H^{+}+e^{-}\right)\;\;\left(E^{\circ }=2.38V_{RHE}\right)$$

(3a) $$*+H_{2}O\to OH*+\left(H^{+}+e^{-}\right)\;\mathrm{the}\;\mathrm{same}\;\mathrm{as}\;\;\mathrm{Equation}\;1A$$

(3b) $$OH*\to OH{*}_{\left(\mathrm{aq}\right)}+*$$

**Figure 3:**
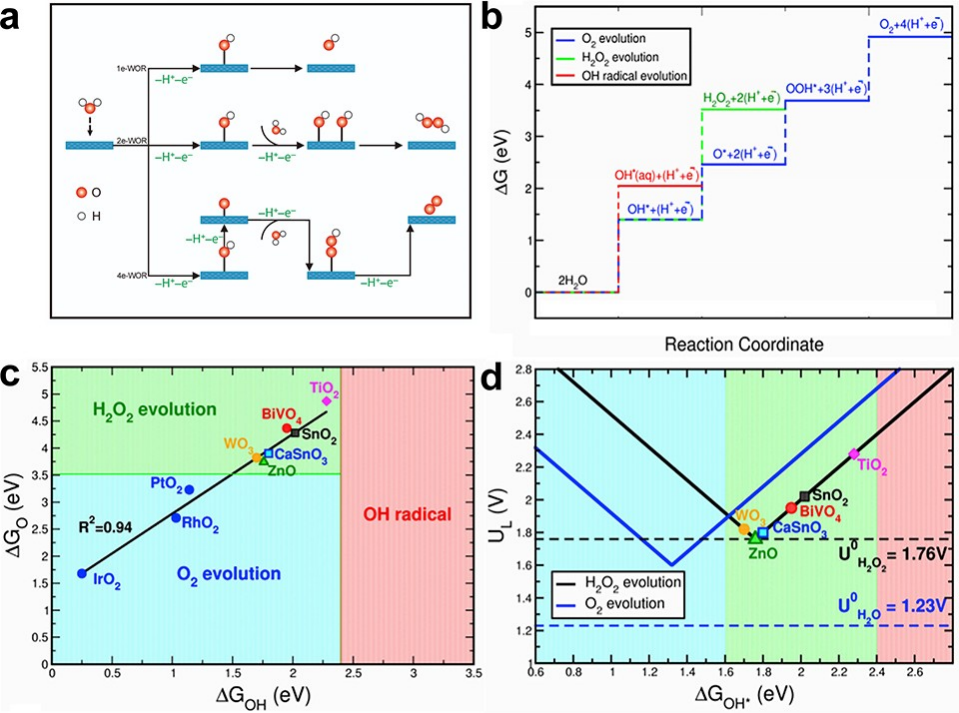
(a) Schematic of three different WOR pathways on the catalytic surface. (b) Free‐energy diagram of three WOR pathways to OH⋅(red), H_2_O_2_ (green), and O_2_ (blue) on a hypothetical ideal catalyst material at U=0 V.^[3]^ (c) Selectivity map among three WORs based on thermodynamic analysis of binding free energies of ΔG_O*_ and ΔG_OH*_.^[3]^ (d) Activity map for 2e‐ and 4e‐WORs. The blue and black lines are the calculated limiting potential U_L_ for 2e‐ and 4e‐WOR, respectively. Reprinted (adapted) with permission from ref. [3] Copyright 2021 Elsevier.

The 2e‐WOR (Figure 3a) is thermodynamically unfavorable for its higher equilibrium potential (E°=1.76 V vs. RHE). It is inherently more difficult to selectively produce H_2_O_2_, as it requires a voltage that is 530 mV higher than that required for producing oxygen. (Equation 2). The most extensively researched WOR pathway is the 4e‐WOR (as shown in Equation 1), 4e‐WOR is thermodynamically favorable because it has the lowest equilibrium potential (E°=1. 23 V vs. RHE) for generating O_2_, which is known as the OER. In addition, H_2_O_2_ can spontaneously decompose into H_2_O and O_2_ or undergo free radical cleavage, resulting in H_2_O_2_ and OH⋅.

### 2.2 Dynamics of 2 e‐WOR

The stepwise mechanism of H_2_O_2_ synthesis by 2e‐WOR can be clarified using theoretical calculations, which can also reveal possible intermediates and transition states. The binding free energy of intermediates, such as ΔG_O*_; ΔG_OH*_; and ΔG_OOH*_, is calculated using DFT to comprehend the mechanism of the electrochemical oxidation process (Figure 3b). The initial stage of the oxidation reaction is the formation of OH* species on the catalyst surface, which occurs on all three WOR routes. The OH* is a critical intermediate, and the ΔG_OH*_ determines the oxidation product. As a result, the electrocatalyst must be able to break the HO−H bond and release OH* from surface. It is energetically advantageous to release OH* into solution as OH⋅ when the ΔG_OH*_ is more than the production energy of an aqueous hydroxyl radical, (ΔG_OH*_>2 : 38 eV; 1e^−^WOR). Conversely, if ΔG_OH*_<2.38 eV, the adsorbed OH* can undergo further oxidation, leading to the production of adsorbed O* or H_2_O_2_. The formation of H_2_O_2_ (2e‐WOR) will be favored if ΔG_O*_ is greater than 3.52 eV; otherwise, the evolution pathway of O_2_ will be favored (4e‐WOR). Thus, the adsorption strength of OH* and O* on the surface, which determines the WOR selectivity (Figure 3c).

Electrocatalytic activity is usually described by the limiting potential (U_L_), which refers to the minimum potential at which all the WOR reaction steps in the free energy diagram become energetically favorable.^[67]^ The limiting potentials (U_L_) for the WOR can be expressed as a function of ΔG_OH*_ since the O* and OOH* intermediates are linear scaled related. (ΔG_O*_=2ΔG_OH*_+0.28 eV; ΔG_OOH*_=ΔG_OH*_+3.2 eV).^[69]^ For 2e‐WOR, the overpotentials (η_H2O2_) and the limiting potentials (U_L_) are determined by using ΔG_OH*_ (Equation 4 A and B) as follows:

(4a) $$U_{L,H_{2}O_{2}}=\left[\max\left(\Delta G_{OH*},\;1.76eV-\Delta G_{OH*}\right)\right]/\;e$$

(4b) $$\eta_{H_{2}O_{2}}=U_{L,H_{2}O_{2}}-1.76eV$$

Figure 3d shows the volcano plots of $U_{L,\;H_{2}O_{2}}$ and $U_{L,\;O_{2}}$ as a function of ΔG_OH*_. When $U_{L,\;H_{2}O_{2}}$ <$U_{L,\;O_{2}}$ , 4e‐WOR is thermodynamically favored over 2e‐WOR. When the ΔG_OH*_ is between 1.6 and 2.4 eV, the WOR is preferred to 2e‐WOR pathway, which lead to the H_2_O_2_ production. These thermodynamic guidelines construct a selectivity map and can be used to search for selective and active catalysts for H_2_O_2_ production.^[3]^

### 2.3 Stability of Catalysts

The 2e‐WOR reaction conditions subject catalysts to a higher oxidation potential exceeding 1.76 V vs. RHE, thus, a crucial element of the reaction is the catalyst′s electrochemical stability. It is well known that the stability is significantly influenced by the pH value of the electrolyte as well as by the potential. Transition metal oxides are mostly concerned materials because of their greater tolerance to oxidative environments. For guiding experimental research, the Pourbaix diagram could be used to find stable electrocatalysts for 2e‐WOR. Pymatgen^[71]^ and Materials Project^[72]^ can be used to construct the Pourbaix diagram, which is an excellent thermodynamic tool for predicting the electrochemical stability of catalysts. It should be noted that the Pourbaix diagram does not consider dynamics.

**Figure 4:**
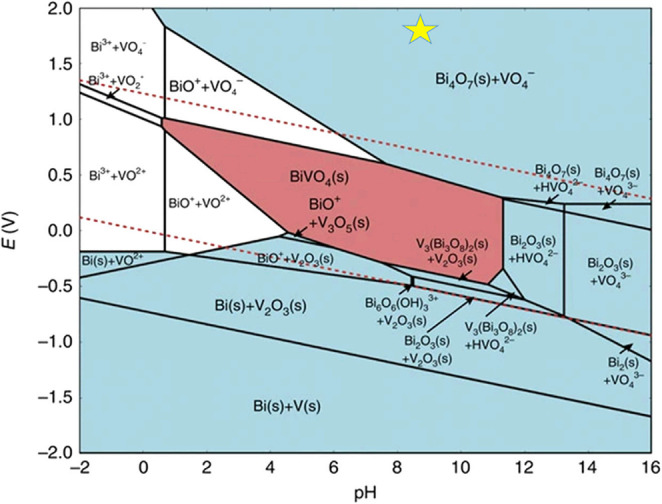
The Materials Project Pourbaix Diagram of 50 %–50 % Bi−V System in Aqueous Solution, Assuming a Bi‐Ion Concentration of 10^−5^ mol/kg and V–Ion Concentration of 10^−5^ mol/kg. Reprinted (adapted) with permission from ref. [3] Copyright 2021 Elsevier. Reprinted (adapted) with permission from ref. [33] Copyright 2016 Nature Publishing Group. (The upper and lower dashed lines correspond to the equilibrium potentials for the OER and HER, respectively. The blue regions denote stable solid compounds, while the pink region denotes the pH and potential window in which BiVO_4_ is stable. The yellow star denotes the experimental conditions for 2e‐WOR in 2 M KHCO_3_, a typical electrolyte for 2e‐WOR.)

BiVO_4_ possess high catalytic activity for 2e‐WOR, but as observed by Toma et al.^[73]^ it has poor electrochemical stability. BiVO_4_ is only stable in the low potential range of 0.3–1.1 V_RHE_ in the pH range (~8.3) of bicarbonate‐based electrolytes commonly used in 2e‐WOR studies, while vanadium oxides (VO_4_^−^) dissolve at higher potentials. The hybrid computational and experimental Bi−V Pourbaix diagram shows different regions, including complete dissolution, solution phase species, partial dissolution, surface passivation, and decomposition into other solid phases (Figure 4). The kinetic and thermodynamic formulations of electrocatalytic reactions are closely related to each other.^[74]^

High‐throughput computational data help to predict novel catalyst materials. Very recently, a structural engineering procedure has been demonstrated to improve the efficiency of 2e‐WOR. Baek et al. investigated the importance of stability in selecting the best perovskites for 2e‐WOR from more than 2000 ABO_3_ structures by using three stability parameters.^[7]^ The application includes approximately 560 distinct ABO_3_ cubic perovskites depicted. The adsorption energies of OH*, O*, and OOH* on all ~560 ABO_3_ perovskites are included in this research. The specific filters are shown below:

1. Thermodynamic stability. It is desirable to quickly filter potential materials for 2e‐WOR by considering the known thermodynamic analysis plots. The decomposition energy, which is the difference between the Gibbs free energy of the catalyst and the most stable product, is the amount of energy required to decompose a catalyst in an aqueous medium to its most stable product. According to Singh et al., the decomposition energy must be less than 0.1 eV to ensure maximum thermodynamic stability of perovskites.^[75]^
2. Electrochemical stability. Pourbaix Diagram Analyzer is used in conjunction with Python Material Genome (Pymatgen) and Materials Project to evaluate stability under electrochemical conditions.[^71^, ^72^]
3. Screening space group. Materials with a larger A cation and cubic Pm3m space group crystal structures, which are the most stable and typical, are selected.[^6^, ^66^, ^76^]

The above methods for evaluating stability can help us in the screening and designing of efficient catalysts which possess durability at high oxidation potentials. Using a vast data set of structures, this descriptor‐based approach offers a simple way to quickly identify the most promising catalyst materials. This will direct the rational catalyst design description that follows.

## 3 Standardized Assessment of Catalyst Performance via 2 e‐WOR

### 3.1 Quantification of H_2_O_2_

One of the most important metrics for assessing the effectiveness of 2e‐WOR catalysts is to precisely measure the H_2_O_2_ content in the electrolyte, it is a challenging task to measure H_2_O_2_ accurately. The chemical instability of H_2_O_2_ may be impacted by complex factors, so it is essential to have a comprehensive and accurate quantification procedure.^[77]^ Currently available practical techniques include fluorescence,^[78]^ chemiluminescence,^[79]^ spectrophotometry, titration,^[80]^ and colorimetric test paper.[^48^, ^74^] The two categories of quantitative H_2_O_2_ approaches are cumulative analysis and in situ analysis.

In situ catalyst performance evaluations are straightforward and intuitive, and it can be supplemented by quantitative methods based on accumulation.

**Figure 5:**
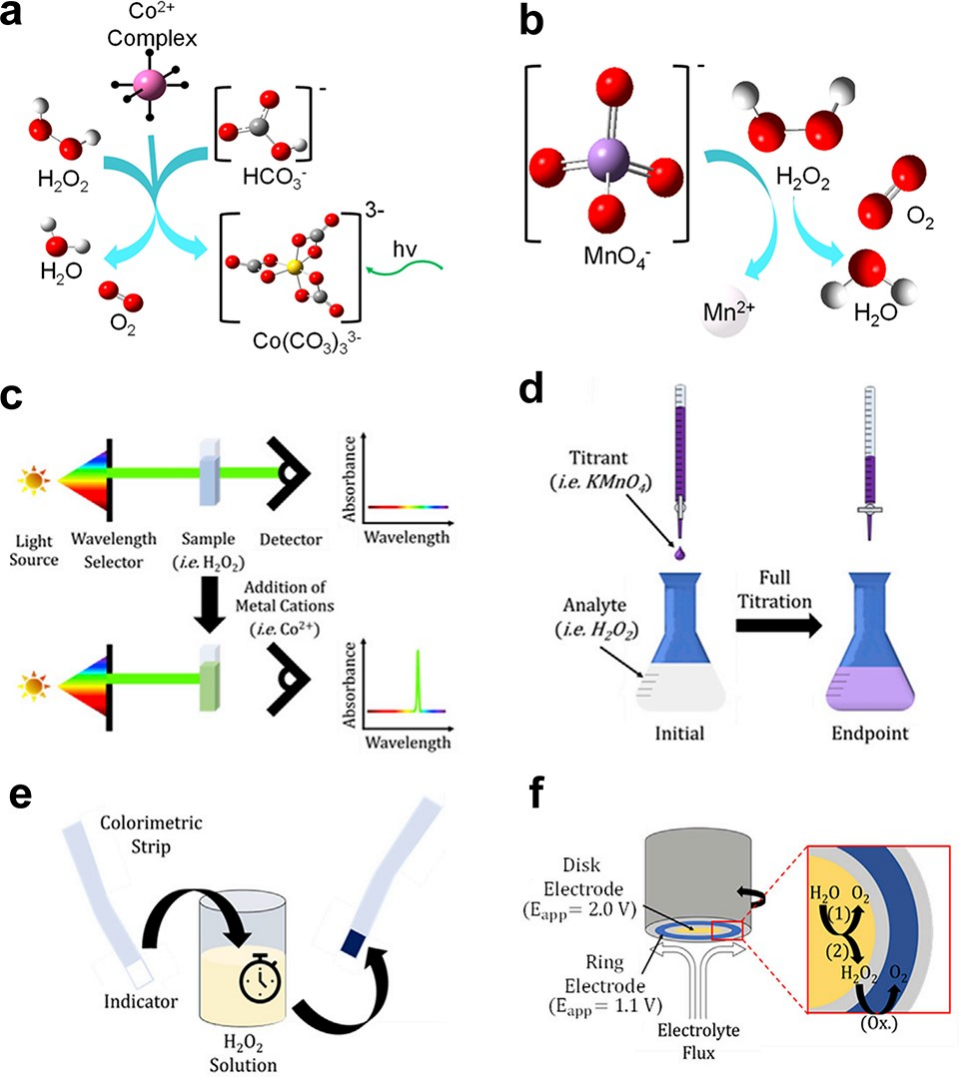
(a) The reaction mechanism of UV‐vis quantifying H_2_O_2_ method using the Co/Co_3_ assay.^[77]^ (b) The mechanism of KMnO_4_ reduction for titration method. Reprinted (adapted) with permission from ref. [37] Copyright 2020 American Chemical Society. (c–f) Schematic represented accumulation and in situ quantification methods of H_2_O_2_. Reprinted (adapted) with permission from ref. ^[3]^ Copyright 2021 Elsevier.

The advantage of in situ methods is that they avoid H_2_O_2_ degradation during prolonged measurements,[^3^, ^81^] but accumulation‐based approaches more reflect the amounts of created H_2_O_2_ seen in a static electrolyzer system.^[6]^ Two concentric electrodes are positioned on a cylindrical shaft in RRDE tests (Figure 5f). The disk electrode linearly swept through the 2e‐WOR region. As the shaft rotates, laminar electrolyte flows from the inner disk electrode, which is where water oxidation (converting H_2_O to H_2_O_2_ and O_2_) takes place, to the outer ring electrode, which is where the produced peroxide is selectively oxidized (converting H_2_O_2_ to O_2_).^[82]^


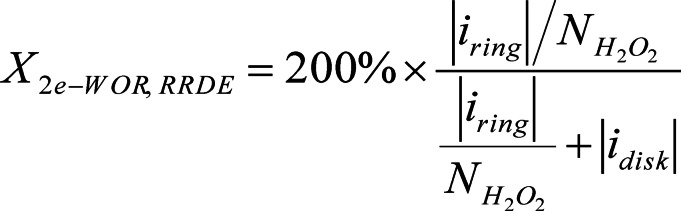




In the given Equation (5), X_2e‐WOR, RRDE_ is expressed as a percentage; i_ring_ represents the measured ring current; $N_{H_{2}O_{2}}$ signifies the H_2_O_2_ collection efficiency; and i_disk_ stands for the measured disk current.^[83]^ While 2e‐ORR investigations have mostly used this equation, it should be noted that the identical equation may be obtained for 2e‐WOR, assuming negligible ⋅OH production.[^46^, ^84^] Xia et al. distinguished between reporting X_2e‐WOR, RRDE_ (Equation 6) and faradaic efficiency (FE_2e‐WOR, RRDE_) as the principal H_2_O_2_ selectivity metric in RRDE studies.^[86]^

It contends that Equation 6′s validity is impacted by side reactions. The authors recommend employing FE_2e‐WOR, RRDE_ as the main parameter for reporting H_2_O_2_ selectivity in RRDE examinations in light of these factors. In addition, there are quantification approaches based on accumulation. The common techniques are as follows:

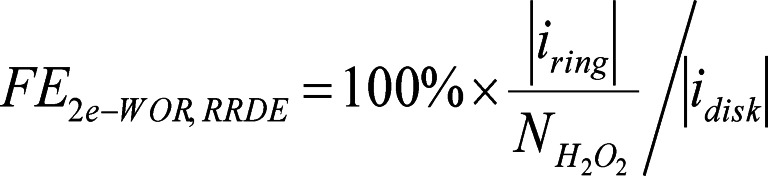




Spectrophotometry: The technique basically depends on a sample concentration, for which the precise H_2_O_2_ concentration is known to be used as a calibration.^[87]^ H_2_O_2_ complexing agents based on iron,^[89]^ copper,^[90]^ and cobalt carbonate are frequently employed in spectrophotometry (Figure 5a).[^58^, ^66^, ^91^] Precise quantitative findings can be achieved by comparing the absorbance of Co^3+^ at 257 nm between the product and the electrolyte mixture after complexation for 30 minutes. Within a specific pH range, a relative inaccuracy of 5 % can be attained. The range of the minimum detection limits is 0.3–4 ppm (Figure 5c).^[92]^

Titration: The titration is a straightforward, convenient procedure with good precision which doesn′t require any specific equipment or calibration (Figure 5d). It is the most widely used H_2_O_2_ quantitative analysis method, and KMnO_4_ and CeSO_4_ are the most commonly used quantification systems.[^6^, ^48^, ^95^] However, the stability time of color changes is also controversial and prone to error.

Colorimetric test paper: Colorimetric test paper can be used without the need for extra equipment or the creation of a standard curve prior to measurement (Figure 5e).^[66]^ By submerging the test paper in an H_2_O_2_ solution for a predetermined amount of time and allowing it to react chemically to change color, one can approximately calculate the yield.^[98]^ It is only appropriate for rough quantification. Aside from the product H_2_O_2_, there are other elements that might result in color changes, thus, there is a lack of accuracy and the detection findings often exceed the actual data.

Every technique has benefits and drawbacks of its own. Gaining a thorough understanding of each method′s operation, factors, and application conditions is highly beneficial for accurately measuring the H_2_O_2_ content. Zheng et al. reported standard operating procedures for the three techniques in H_2_O_2_, and compared each method′s time stability, sensitivity to pH, interference effect, and other features.^[77]^ Ultimately, the accuracy of each measurement method was obtained in the concentration range of 5–1000 ppm. The average relative error of the spectrophotometer method in the range of 5–1000 ppm is less than 5 %, which is the best quantitative method of the three.

The error levels between titration and spectrophotometry procedures are generally equal when the concentration of H_2_O_2_ is more than 150 ppm.

The KMnO_4_ titration method employed in 2e‐WOR exhibits a delayed response time, is vulnerable to over‐titration, and may result in gas production, which could cause analyte overflow and subsequent measurement inaccuracies. The KMnO_4_ titration method frequently overestimates the production of H_2_O_2_. The accuracy of the titration decreases with decreasing H_2_O_2_ concentration. It was hypothesized that the inaccuracy resulted from the end point′s hazy color differentiation. However, this method continues to be a reasonably reliable and stable analytical technique when the quantification procedure is not affected by interferences like acetone, chlorates, or possible nitrates.^[86]^ It is widely utilized because of its wide application among the electrolytes frequently used for H_2_O_2_ measurement and its insensitivity to pH fluctuations.

A description of the techniques for detecting hydrogen peroxide is given in Table 1. H_2_O_2_ produced electrochemically requires careful preparation of the calibration and the avoidance of elements that could interfere with the assay before measurement. Based on the findings, the aforementioned techniques all have a quick detection time and can measure H_2_O_2_ concentrations within the proper pH and measurement ranges (the stability is negligible). Although the colorimetric test paper method has irreplaceable convenience, it is unable to accurate quantification, such as color sensitivity following air exposure and the impact that various solvents have on color development. Over the whole 5–1000 ppm range, the colorimetric test paper shows a relative inaccuracy of more than 10 %.^[77]^

**Table 1:** A summary of H_2_O_2_ Measurement Techniques.

Methods	Advantages	Disadvantages
RRDE	H_2_O_2_ selectivity metric	electrochemical equipment needed
Titration	no equipment needed, accurate quantification over a wide concentration range: 1,000–10,000 (ppm)	endpoint ambiguity, low detection sensitivity
Spectro‐ photometry	high detection sensitivity: 0.3–4(ppm)	susceptible to interference, calibration needed
Colorimetric strips	Convenience no equipment needed	time‐sensitive poor accuracy

### 3.2 2 e‐WOR Electrochemical Performance Evaluation Factors

Catalysts’ electrochemical performance is mainly assessed in terms of activity, selectivity, and stability.^[99]^ Activity is one of the most important factors in evaluating the performance of a catalyst. Electrocatalytic activity is usually described by the overpotential and tafel slope. The overpotential typically represents the voltage required to surpass the thermodynamic limit (1.76 V vs RHE), which refers to the minimum potential at which all the reaction steps for WOR become energetically favorable in the calculated free energy diagram. The variation of the electrochemical reaction rate with electrode overpotential is explained by the relationship between tafel slope and overpotential. Two methods are used to measure the electrocatalytic activity in 2e‐WOR: cyclic voltammetry (CV) and linear‐sweep voltammetry (LSV). Firstly, the current density being measured should be purely faradic and only attributable to H_2_O_2_ generation. Therefore, before measuring the overpotential, cyclic voltammograms should be conducted between the non‐faradic region and potentials higher than the maximum voltage in the LSV. It ought to continue operating until the characteristic change from cycle to cycle is minimal. The “activation cycling” procedure ensures the electrocatalyst is oxidized to its operando state.^[100]^ This stage is especially crucial for 2e‐WOR since almost all transition metals can be oxidized by the high anodic bias required to produce H_2_O_2_.^[101]^ Lastly, in order to eliminate solution and ulterior circuitry resistances, all LSVs should be iR‐corrected for the electrochemical cell′s uncompensated resistance.

Selectivity, as one of the most important factors in evaluating catalyst performance, reflects the electrolytic performance and application potential of the catalyst, with higher selectivity catalysts being able to preferentially produce the target product. FE_H2O2_ or RRDE can be used to evaluate the selectivity of the 2e‐WOR. Chronoamperometry (CA) is a technique that is commonly employed in accumulation‐based methods for the evaluation of FE at a given potential. The most widely used descriptor to assess catalyst selectivity in accumulation‐based techniques is the faradic efficiency, which is defined as the ratio of the amount of H₂O₂ generated to the theoretically generated H₂O₂ based on the recorded current.

(7) $$FE_{2e-WOR}=\frac{\mathrm{Amount}\;\mathrm{of}\;\mathrm{generated}\;H_{2}O_{2}}{\mathrm{Theoretically}\;\mathrm{generated}\;H_{2}O_{2}}\times 100\%=\frac{M_{H_{2}O_{2}}}{\frac{\int_{0}^{t}I\left(t\right)dt}{nF}}\times 100\%$$

In the above Equation (7), the M_H2O2_ is the molar amount of generated H_2_O_2_ (moles); I(t) is the measured current as a function of time under the given applied bias (A); t is the duration over which the measured H_2_O_2_ is accumulated (s); n is 2 as two electrons are needed to form one molecule of H_2_O_2_; F is Faraday′s constant (96,485 C/mol). Note that results in FE_2e‐WOR_ expressed as a percentage. Due to its sensitivity to the applied bias, the FE_2e‐WOR_ needs to be evaluated at various potentials, typically in the range of 1.76–4.0 V vs. RHE.^[3]^

Calculating FE_2e‐WOR_ is straightforward, determining M_H2O2_ is challenging due to H_2_O_2_′s poor stability under electrochemical conditions. Disproportionation of H_2_O_2_ can result in the production of H_2_O and O_2_, and it may occur in the electrolyte or on the electrodes. These reactions are affected by the electrolyte species,^[1]^ pH catalyst purity,[^1^, ^4^, ^66^, ^102^] and H_2_O_2_ concentration. Moreover, the accumulation of H₂O₂ in the electrolyte can alter selectivity by disrupting the balance of the electrochemical micro‐environment. To avoid gradients in H₂O₂ concentration occurred on the electrode, a thorough mixing of the electrolyte through pumping or stirring should be ensured during chronoamperometry (CA) tests. This reduces the impact of H₂O₂ accumulation on local equilibrium and enhances the accuracy of accumulation‐based H₂O₂ quantification techniques. To prevent the degradation of H_2_O_2_ by cathodic materials, it is recommended to separate the anode and cathode. Furthermore, to minimize the impact of any remaining concentration gradients, it is suggested that aliquots exceeding 20 % of the total electrolyte volume should be extract to measuring the accumulated H_2_O_2_.^[3]^

Stability, as an important factor in assessing catalyst performance, determines whether the catalysts can be used in practice. Two methods are used to evaluate catalyst stability: chronopotentiometry (CP) and CA. A stable catalyst in 2e‐WOR will usually show no obvious current decay for a long time under a constant applied bias. It is important to periodically refresh the electrolytes to prevent the accumulation of H_2_O_2_.^[3]^

In the performance evaluation of catalysts, discussing the yield (M_H2O2_) is crucial, as the concentration of products accumulated in the reaction directly impacts the catalyst′s performance. Yield is related to catalyst efficiency, selectivity, economics, lifespan, and process optimization. In industrial applications, high yields correspond to greater productivity and lower costs per unit of production. High yields also reduce the generation of waste and by‐products, thereby lessening environmental impact. Variations in yield can indicate the stability and longevity of the catalyst. Consistently high yields often suggest good catalyst durability, while decreasing yields may indicate catalyst deactivation.

It is necessary to describe the partial current density towards H_2_O_2_ (J_H2O2_) as a function of potential,^[4]^ as the side reaction (4e‐WOR) also contributes to the total current at a given potential. To obtain J_H2O2_, the potential dependent FE_2e‐WOR_ can be multiplied by the total current density. It is important to note that J_H2O2_ is often reported by the normalized geometric area, which is a critical metric for applications.^[103]^ Furthermore, J_H2O2_ can be converted to the molar H_2_O_2_ production rate.

In conclusion, it is critical to provide uniform evaluations for both the precise measurement of H_2_O_2_ generation and the analysis of the 2e‐WOR critical indicators. Some auxiliary characterization methods may help us in selecting catalysts more accurately and efficiently during the catalyst performance evaluation process. These thorough studies facilitate the assessment of catalysts as well as the exploration of other significant 2e‐WOR process.

For example, Zhang et al. conducted operando Raman analysis on BiVO_4_ and BiVO_4_‐Air/V during 2e‐WOR in 1.0 mol L^−1^ NaHCO_3_ electrolyte (pH=8.3).^[104]^ The two Raman peaks at 1336 and 1605 cm^−1^ were attributed as the HCO_3_^−^ stretch of electrolyte. The peak intensities were consistent whatever the applied bias, and almost the same for BiVO_4_ and BiVO_4_‐Air/V photoanodes. The Raman spectra of synthetic BiVO_4_ showed a prominent peak at 830 cm^−1^, which was assigned to the O−O stretch in peroxide and OOH* species. Although absorbed H_2_O was present on synthetic BiVO_4_, the symmetrical hydrogen stretch motion at 3400 cm^−1^ was not observed. This suggests that absorbed H_2_O can be easily converted into the OOH* state. In contrast, the Raman spectra of the BiVO_4_‐Air/V photoanode showed a minor peak at 100 cm^−1^, which could potentially be associated with OH or superoxide species. Additionally, a difference in the symmetrical hydrogen stretch motion was observed for the BiVO_4_‐Air/V photoanode. These results suggest that H_2_O absorbed on BiVO_4_‐Air/V is dissociated to OH*.

To gain a deeper understanding of the reaction mechanism of 2e‐WOR at the electrocatalytic interface, the utilization of in‐situ testing techniques represents a highly efficacious approach, it allows real‐time monitoring of the catalyst′s behavior and reaction mechanism under actual conditions. In situ characterization enables researchers to observe structural changes in the catalyst during the reaction, including alterations in active sites on the surface. It also allows for the direct observation of interactions between the catalyst surface and reactants, such as adsorption and desorption processes. By providing real‐time data on catalyst performance, in situ characterization helps researchers gain a deeper understanding of the reaction mechanism and the catalyst′s role.^[44]^ Many research have used theoretical DFT calculations and experimental results to support the idea that oxygen vacancies (Ov) are what drive the increase of 2e‐WOR performance.

Sun et al. used a bifunctional defective TiO_2‐x_ catalyst produced by plasma to build a two‐sided H_2_O_2_ production system.^[105]^ Within the water oxidation process, TiO_2‐x_ can retain a relatively active surface state for continuous H_2_O‐to‐H_2_O_2_ conversion before 2.2 V. The in‐situ Raman results indicate essentially unchanged Raman peaks (all five signals) for TiO_2‐x_ at potentials negative than 2.2 V. When the applied potential is more than 2.2 V, these signals diminished and even vanished, suggesting that the crystal structure started to become significantly distorted at higher potential. Pristine TiO_2_ exhibits few insignificant Raman characteristic peaks at all potentials in comparison to TiO_2‐x_. Combined the LSV measurements and in‐situ Raman analysis, they identified internal O_V_s and surface distortion as the main possible catalytically active sites of enhanced 2e‐ORR and 2e‐WOR processes on TiO_2‐x_ (101).

Wang et al. presented a CO_2_/carbonate mediation approach to steering the WOR pathway from 4e^−^ to 2e^−^. The WOR pathway to H_2_O_2_ is considered to be mediated by carbonate through the formation of carbonate radical and percarbonate intermediates, according to theoretical calculations coupled with electron paramagnetic resonance and isotope labeling methods.^[106]^ They used the electron paramagnetic resonance (EPR) to test the formation of carbonate radical in order to validate the proposed reaction mechanism. They used 5,5‐dimethyl‐1‐pyrroline N‐oxide (DMPO) as a spin trap and an in‐situ trapping technique to detect the generation of the carbonate radical during electrolysis because of its short lifetime. Compared to the direct mixture of DMPO and before/post‐electrolysis carbonate solution, the solution under electrolysis exhibits a clear four‐line 1 : 2 : 2 : 1 splitting pattern characteristic of the DMPO⋅‐OH adduct. This might mean that carbonate radical was formed at the surface of FTO electrode under oxidation potentials. Furthermore, they performed an ^18^O isotope labeling experiment to understand the possible formation of HCO_4_^−^ intermediates.

Compared to the extensive research conducted in other electrocatalysis fields such as ORR, CO_2_RR, and NRR, it is noted that in situ characterization studies related to 2e‐WOR fields are few, more work should be focused on this field.^[107]^

## 4 2 e‐WOR Electrocatalysts Progress

Nowadays, a range of catalysts exhibiting 2e‐WOR activity have been discovered, predominantly composed of metal oxides, specifically noble‐metal‐free transition metal oxides. However, the efficiency of pristine nonprecious metal oxides is relatively low to satisfy the requirement of industrial production. Carbon‐based materials demonstrate exceptional electrical conductivity and electrochemical stability. Carbon‐based materials have cost‐ effective, plentiful, and versatile characteristics. The catalyst′s atomic and electronic structure could be precisely controlled in order to optimize the adsorption energy of reactive intermediates, hence improving the activity, selectivity, and stability of the 2e‐WOR process. Carbon‐based materials are regarded as potential electrocatalysts for 2e‐WOR in the formation of H_2_O_2_, and has receive more and more attention over the last few years. In the Table 2, we list representative studies that have significantly advanced in the field of electrocatalytic anodic H_2_O_2_ production. Various material design and engineering strategies have been developed to improve the performance of electrocatalysis, including as defect engineering, doping engineering, facet engineering, and interface engineering. For example, we can regulate the atomic and electronic structure of metal oxides for optimal intermediates adsorption by either introducing exotic atoms/ions (doping engineering) or extracting original atoms/ions from (defect engineering) the pristine material. Additionally, modulating the surface/interfaces by exposing specific crystal planes (facet engineering) or creating new active interfaces (interface engineering) is also powerful to tune the binding of reactive intermediates toward enhanced 2e‐WOR activity. This section focuses on examining the latest developments in 2e‐WOR electrocatalysts composed of carbon‐based materials and metal oxides, covering design strategies, electrocatalytic performance, structural characterization, and investigations into catalytic mechanisms.

**Table 2:** Performances of 2e‐WOR electrocatalysts in recent years.

Electrocatalyst	Substrate	ConcentrationH2O2/VH2O2	FE	Electrolyte
**unsorted metal oxides**				
MnO_x_ ^[64]^	Au	0.08 μM	77 %@0.59 V vs Ag/AgCl	Butyl ammonium bisulfate
CaSnO_3_ ^[98]^	FTO	4.2 μmol min^−1^ cm^−2^	76 %@3.2 V vs RHE	2 M KHCO_3_
CuWO_4_ ^[112]^	CC	∼ 11.8 μmol min^−1^ cm^−2^	58 %@2.4 V vs RHE	2 M KHCO_3_
CoO^[89]^	FTO	3 μM	–	KHCO_3_
La_2_O_3_ ^[89]^	FTO	∼29 μM	–	KHCO_3_
Nb_2_O_5_ ^[89]^	FTO	∼32 μM	–	KHCO_3_
V_2_O_5_ ^[89]^	FTO	∼36 μM	–	KHCO_3_
Bi_2_O_3_ ^[89]^	FTO	∼ 16 μM	–	KHCO_3_
Al_2_O_3_ ^[89]^	FTO	46 μM	–	KHCO_3_
TiO_2_ ^[89]^	FTO	73 μM	–	KHCO_3_
BiVO_4_ ^[89]^	FTO	70 mM (1.8 C)	35 %@3 V vs Ag/AgCl	KHCO_3_
WO_3_ ^[66]^	FTO	0.5 mM min^−1^	47 %@2.25 V vs RHE	NaHCO_3_
SnO_2_ ^[66]^	FTO	∼ 1.5 μM min^−1^	50 %@3.1 V vs RHE	NaHCO_3_
BiVO_4_ ^[66]^	FTO	∼6 mM min^−1^	70 %@3.1 V vs RHE	NaHCO_3_
TiO_2_ ^[66]^	FTO	∼ 1 μM min^−1^	19 %@3.25 V vs RHE	NaHCO_3_
LaAlO_3_ ^[7]^	FTO	0.16 mmol cm^−2^	87 %@3.34 V vs RHE	2 M NaHCO_3_
ZnGa_2_O_4_ ^[113]^	Pt foil	0.26 μmol cm^−2^	82 %@2.3 V vs RHE	2 M KHCO_3_
Ru_x_Ti_1–x_O_2_ ^[114]^	Carbon paper	24.2 μmol cm^−2^	62.8 %@>100 mA cm^−2^	2 M KHCO_3_
Ni_x_Ti_1–x_O_2–y_ ^[115]^	Carbon paper	32.7 μmol cm^−2^ @3.2 V vs RHE	70 %@~100 mA cm^−2^	2 M KHCO_3_
Pr_1.0_Sr_1.0_Fe_0.75_Zn_0.25_O_4–δ_ ^[116]^	carbon cloth	–	~80 %@2.0 V vs RHE	0.5 M KHCO_3_
**Defect engineering of metal oxides**				
CaSnO_3_@CF^[5]^	Carbon fiber	39.8 μmol min^−1^ cm^−2^	∼90 %@2.9 V vs RHE	2 M KHCO_3_
ZnO@CF^[117]^	Carbon fiber	19.7 μmol min^−1^ cm^−2^	83.8 %@2.8 V vs RHE	2 M KHCO_3_
TiO_2–x_ ^[105]^	FTO	20 mmol L^−1^ h^−1^	40 %@2.2 V vs RHE	2 M KHCO_3_
WO_3_/SnO_2–x_ ^[118]^	FTO	0.2 mmol h^−1^ cm^−2^	80 %@ 3.2 V vs. RHE	2 M KHCO_3_
**Facet control engineering of metal oxides**				
ZnO (1010)^[4]^	FTO	–	81 %@2.6 V vs RHE	2 M KHCO_3_
BiVO_4_ (010)^[119]^	FTO	19.7 μmol min^−1^ cm^−2^	83.8 %@2.8 V vs RHE	2 M KHCO_3_
**Doping engineering of metal oxides**				
Gd:BiVO_4_ ^[74]^	FTO	35 μmol min^−1^ cm^−2^	78 %@3.1 V vs RHE	K_2_CO_3_
C,N:TiO_2_ ^[120]^	Ti sheet	0.29 μmol L^−1^ cm^−2^ h^−1^	8 % @ 2.9 V vs Ag/AgCl	0.05 M Na_2_SO_4_
Bi_2_WO_6_:Mo^[121]^	FTO	300 μmol h^−1^ cm^−2^	79 %@3.2 V vs RHE	KHCO_3_
**Defect engineering of carbon fiber materials**				
CNFs/NF‐600^[122]^	Ni foam	22.6 μmol min^−1^ cm^−2^	41.2 %@2.9 V vs RHE	1 M Na_2_CO_3_
CNFs/NF@PTFE‐60 %^[122]^	Ni foam	19.7 μmol min^−1^ cm^−2^	53.8 %@2.8 V vs RHE	1 M Na_2_CO_3_
WO_3–X_/CNTs@CF‐600–2^[123]^	FTO	10.3 μmol min^−1^ cm^−2^	54 %@2.48 V vs RHE	2 M KHCO_3_
**Interface engineering of CFP**				
Carbon fiber paper(CFP)^[124]^	–	190.9 μmol L^−1^	3.1 %@1.5 V vs Ag/AgCl	–
Carbon cloth (CC)^[124]^	–	117.2 μmol L^−1^	1.9 %@2.0 V vs Ag/AgCl	–
Gas diffusion layer (C‐GDL)^[124]^	–	87.3 μmol L^−1^	1.4 %@2.2 V vs Ag/AgCl	–
Carbon felt (CF)^[124]^	–	83.5 μmol L^−1^	1.3 %@1.3 V vs Ag/AgCl	–
Glassy carbon (GC)^[124]^	–	82.0 μmol L^−1^	1.3 %@2.1 V vs Ag/AgCl	–
PTFE/CFP^[6]^	GC	23.4 μmol min^−1^ cm^−2^	66 %@640 mV	1 M Na_2_CO_3_
CFP(SAMs)^[8]^	CFP	79.8 μmol min^−1^ cm^−2^	82.5 %@2.1 V vs RHE	3.5 M KCl
**Boron‐doped diamond (BDD)**				
BDD‐UNC^[125]^	–	0.13 mM	–	0.12 M Ti(SO_4_)_2_
BDD^[126]^	Ti	19.7 μmol min^−1^ cm^−2^	28 %@3.5 V_RHE_	1.0 mol dm^−3^ KHCO_3_
BDD/Nb^[127]^	–	0.3–0.5 mM	–	0.25 M HClO_4_
Si/BDD^[128]^	Si	0.55 mM	–	0.05 M Na_2_SO_4_
Nb/BDD microfilms^[63]^	–	76.4 μmol min^−1^ cm^−2^	87 %@2.85 V_RHE_	2 M K_2_CO_3_/KHCO_3_

For the past few years, the primary emphasis of the study has been on oxide materials. The activity of metal oxide materials could be improved with different strategies. An ideal electrocatalyst should meet the requirement of having a low activation barrier for the oxidation of OH* to OOH*. Besides, the electrocatalyst should also have moderate binding with oxygen intermediates, which lead to a high selectivity. It is possible to improve the electrocatalytic performance of metal oxides by strategically designing the active sites at the molecular level. The efficacy of metal oxide based electrocatalysts is influenced by several factors, such as surface morphology, crystal structure, and oxidation state. The work function of the surface of metal oxide could be modified by introducing an electron donor or acceptor, which alters the electron affinity energy and surface energy. Furthermore, the introduction of ligands leads to a coupling between the d‐orbitals of the ligands and the electronic states of the metal oxide surface. This coupling results in charge transfer or polarization, which can induce alterations in the active site and impact the 2e‐WOR. The following are a few fundamental design strategies for metal oxide catalysts.

### 4.1 Defect Engineering of Metal Oxides

Defect engineering is a useful method to regulate the electronic property of the metal oxide, which has a significant effect on the catalytic activity.^[108]^ Defect‐rich metal oxides often have more structural distortion, fewer coordination numbers, and a large number of dangling bonds on their surface. These characteristics can significantly affect the catalytic performance of defected metal oxides.^[109]^ Among various defects, oxygen vacancy (O_V_) is the most well studied which can not only regulate the oxidation states of metal centers but also moderate the absorption energy of intermediates as well as introduce new active sites into the catalysts.^[111]^

**Figure 6:**
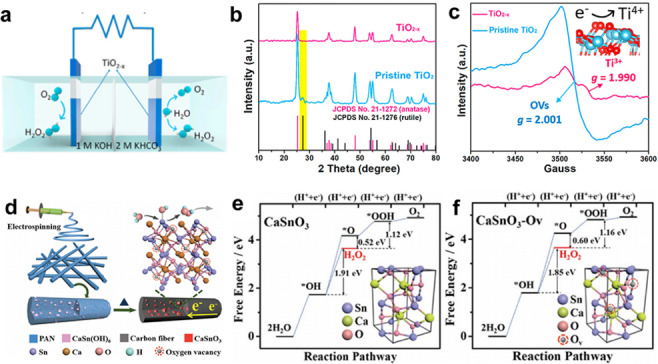
(a) Schematic illustration of the tandem cell using TiO_2–x_ as the bi‐functional electrode. (b) XRD tests of pristine TiO_2_ and TiO_2–x_. (c) EPR spectra of pristine TiO_2_ and TiO_2–x_. Reprinted (adapted) with permission from ref. [59] Copyright 2021 Elsevier. (d) Schematic representation of the synthetic procedures of CaSnO_3_@CF and oxygen vacancy promoted 2e‐WOR process. (e–f) DFT free energy diagram of CaSnO_3_ and CaSnO_3_‐O_v_. The insets are structural illustrations of CaSnO_3_ and CaSnO_3_‐O_v_ without and with oxygen vacancy, respectively. Reprinted (adapted) with permission from ref. [5] Copyright 2021 John Wiley and Sons.

Dong et al. used a hydrogen plasma etching technique creating oxygen vacancy in TiO_2_ to control surface characteristics and electronic structures, and suggested an electrochemically driven H_2_O_2_ production system (2e‐ORR/2e‐WOR cell) using TiO_2‐x_ as bifunctional electrocatalysts.^[105]^ Figure 6a shows the construction of the coupled electrolytic cell. During the catalytic process, in situ electrochemical Raman spectroscopy was used to clarify the catalytic mechanism of 2e‐WOR on the surface of TiO_2_. Surface rebuilding takes place during the water oxidation process. Therefore, this implies that surface distortion at particular 2e‐WOR operating voltages may be responsible for the electrocatalytic activity of TiO_2–x_ for H_2_O_2_ production. When comparing TiO_2–x_ to the original TiO_2_, X‐ray diffraction (XRD), X‐ray photoelectron spectroscopy (XPS) analysis and electron paramagnetic resonance (EPR) measurements showed a notable rise in the oxygen defect content. The introduction of flaws is the cause of this growth. Compared with TiO_2–x_, pristine TiO_2_ exhibits few insignificant Raman characteristic peaks at all applied potentials, and more importantly, the obvious surface reconstruction can be found for pristine TiO_2_ when the potential increases from 2.2 V–2.4 V (Figure 6b–c). It can be concluded that crystal lattice disorder of TiO_2–x_ may be the activity source of 2e‐WOR. In a word, the internal O_V_ and surface distortion are the main possible catalytically active sites for enhanced 2e‐ORR and 2e‐WOR processes of TiO_2–x_, respectively.

Because of this, TiO_2–x_ showed more selectivity for 2e‐WOR to create H_2_O_2_, with the maximum FE_H2O2_ at 2.1 V vs. RHE being roughly 55 %. Using 1 mol L^−1^ KOH electrolyte, the cathode attained 2e‐ORR, and the anode, using 2 mol L^−1^ KHCO_3_ electrolyte, obtained 2e‐WOR. This interaction yielded H_2_O_2_, with an impressive FE_H2O2_ of 134.2 % and a high yield of about 20 mmol L^−1^ h^−1^.

As Shown in Figure 6d, Zhang et al. have prepared CaSnO_3_ nanoparticles encapsulated in electrospun carbon fibers (CaSnO_3_@CF).^[5]^ The XPS O_1s_ spectra of both CaSnO_3_ and CaSnO_3_@CF indicated the content of O_V_ in CaSnO_3_@CF is higher than that of CaSnO_3_, which is confirmed by EPR results. The prepared CaSnO_3_@CF exhibits the highest H_2_O_2_ generation rate of ≈39.8 μmol min^−1^ cm^−2^ and the highest FE of ≈90 % for 2e‐WOR at 2.9 V versus RHE.^[117]^ Also, the authors construct theoretical models of CaSnO_3_ with/without O_V_ to explore the impact of O_V_ on the catalytic performance. According to the DFT result of Figure e–f, the presence of O_V_ can control the Sn atoms in the active metal center to shift from an electron‐deficient state to an electron‐rich configuration, optimizing the adsorption free energy of intermediates. It showed an increase in the energy barrier from H_2_O_2_ to OOH* (from 1.12–1.16 eV) and a decrease in the energy barrier from OH* to H_2_O_2_ (from 1.91–1.85 eV), suggesting that 2e‐WOR is thermodynamically more favorable on an O_V_‐rich surface.

Similarly, Yu et al. used the electrospinning pyrolysis process synthesized ZnO anchored in hollow carbon fiber films (ZnO@CF) for the catalysis of 2e‐WOR.^[117]^ These films showed a hierarchical structure resembling foam made up of O_v_‐rich ZnO nanocrystals and interconnected hollow carbon nanotubes. By maximizing the interaction between oxygen intermediates, the high O_V_ content of ZnO@CF displayed remarkable H_2_O_2_ selectivity. Moreover, a high surface area of the hollow carbon shell revealed more active sites in addition to speeding up the electron transfer capabilities. The free‐standing membrane electrode demonstrated exceptional 2e‐WOR activity with high selectivity (83.8 % at 2.8 V vs. RHE), a high H_2_O_2_ production rate (19.7 μmol cm^−2^ min^−1^) and strong stability.

Dai et al. reported that oxygen‐deficient Pr_1.0_Sr_1.0_Fe_0.75_Zn_0.25_O_4–δ_ chalcogenide oxides (D‐PSFZ) are favorable for binding reaction intermediates and promoting selective and efficient two‐electron transfer pathways.^[116]^ In this work, a new class of oxygen‐deficient chalcogenide oxides (D‐PSFZ) enriched in oxygen vacancies (O_V_) is developed for highly selective H_2_O_2_ generation. These chalcogenide oxides have unique structural tunability at the atomic level (ABO_3_, with an A‐site containing rare earth ions and a B‐site containing transition metal ions), allowing a precise control of electrochemical activity and a deep understanding of structure‐property relationships. Specifically, the researchers observed that the formation of oxygen vacancies (O_V_) activated reactive sites (i. e., Zn and Fe cations), enhancing the binding of HOO* intermediates to prevent the dissociation of HOO* into O* and OH^−^, thereby eliminating the 4e transfer pathway. Under various electrolyte conditions (0.1~2.0 M KHCO_3_), D‐PSFZ exhibited more than 99 % FE over the entire ORR potential interval (0.05–0.45 V vs. RHE) and was stable for 500 h, which is important for scalable H_2_O_2_ electrosynthesis. In addition, the D‐PSFZ exhibited a significantly high 2e‐WOR selectivity of 80.0 % at a lower potential of 2.15 V. With a highly efficient bifunctional D‐PSFZ catalyst serving as both the cathode and anode, a membrane‐free monolithic H_2_O_2_ electrolytic cell can achieve an overall FE of 163.0 % at 50 mA cm^−2^ current density and 2.10 V vs.RHE. It exhibits excellent electrochemical stability with negligible potential fluctuations for 100 h.

### 4.2 Doping Engineering of Metal Oxides

One crucial method for Changing the physicochemical characteristics of catalysts is doping engineering. The electronic structure of the active site can be optimized to enhance the catalyst′s performance by introducing new elements into the structure.^[129]^

The activity trends of 2e‐WOR for four distinct metal oxides (WO_3_, BiVO_4_, TiO_2_, and SnO_2_) were anticipated by Nørskov et al. BiVO_4_ stood out among them as a promising and superior 2e‐WOR catalyst. Particularly, Gd‐doped BiVO_4_ can outperform pristine BiVO_4_ in terms of catalytic stability and selectivity towards H_2_O_2_ production through doping engineering.^[74]^ Gd was chosen as the dopant to strengthen the electrocatalytic performance because of its half‐filled 4 f valence shell. Compared to Bi, which has a strong oxygen bond and raises the energy barrier for the dissolution of VO_4_^3−^ anions, the Gd atom is more oxyphilic. The Gd:BiVO_4_ sample showed a high FE (H_2_O_2_) of 78 % at 3.2 V vs. RHE and a lower overpotential, which was reduced by about 110 mV at 5 mA cm^−2^ with an appropriate doping dosage of 6 %. The various BiVO_4_ facets with different Gd doping were constructed for theoretical calculation, and the Bi surface atoms on these facets are the primary adsorption sites for the oxygen intermediates. The DFT results show that the low Gd−Bi bridges possess more suitable ΔG_OH*_ values for H_2_O_2_ production than the pristine Bi−Bi sites, which suggest a low overpotential can be obtained.

**Figure 7:**
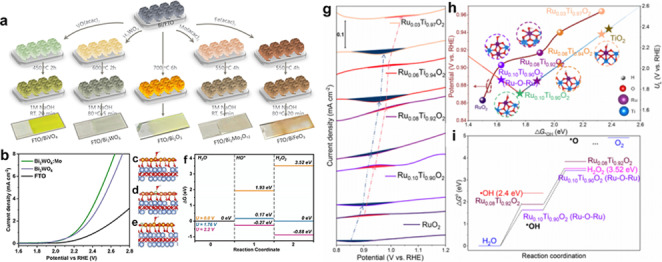
(a) The schematic diagram for the synthesis of Bi‐based transition metal oxide electrocatalysts. (b) LSV curves of Bi‐based transition metal oxides. Structure of OH* on (c) Bi_2_WO_6_, (d) Mo‐Bi_2_W(Mo)O_6_, and (e) MoBi_2_W(W)O_6_. (f) Energy diagrams for the H_2_O_2_ evolution on Mo doped Bi_2_WO_6_. Reprinted (adapted) with permission from ref. [74] Copyright 2020 John Wiley and Sons. (g) CV curves for Ru_x_Ti_1–x_O_2_ and RuO_2_ electrodes collected in 2 mol L^−1^ KHCO_3_ electrolyte at a scan rate of 50 mV s^−1^. The corresponding OH* desorption/adsorption potential peak is marked by an arrow. (h) OH* desorption potential and UL values vs. the calculated G*OH values on the Ru_x_Ti_1–x_O_2_ catalysts. UL is the limiting potential at which the electrochemical reaction spontaneously occurs. (i) Adsorption free energy for *OH and *O intermediates on Ru_0.08_Ti_0.92_O_2_ and Ru_0.10_Ti_0.90_O_2_ calculated through DFT. Reprinted (adapted) with permission from ref. [67] Copyright 2023 Elsevier.

Li et al. prepared various Bi_2_WO_6_ via electrodeposition method and explored their electrocatalytic performance for 2e‐WOR (Figure 7a).^[121]^ The pristine Bi_2_WO_6_ exhibits both high FE and production rate toward H_2_O_2_ generation, reaching 39.7 % and 42.4 μmol min^−1^ cm^−2^, respectively, at 2.6 V versus RHE. The Mo species was doped to further promote the catalytic activity. The Bi_2_WO_6_:Mo demonstrate higher current densities at wide potential range of 1.7–3.2 V versus RHE and higher FE of 79 % at 3.2 V versus RHE, which is superior to that of pristine Bi_2_WO_6_ (Figure 7b). To gain a better understanding of the 2e‐WOR mechanism on the surface of Bi_2_WO_6_:Mo, three different models were constructed, which are Bi_2_W(W)O_6_ (OH intermediate locates near the W site of pure Bi_2_WO_6_), Bi_2_W(Mo)O_6_:Mo (OH intermediate locates near the Mo site of Mo‐doped Bi_2_WO_6_), and Bi_2_W(W)O_6_:Mo (OH intermediate locates near the W site of Mo‐doped Bi_2_WO_6_),respectively. DFT calculations reveal that the Gibbs free energy change of *OH (ΔG_*OH_) over Bi_2_W(W)O_6_:Mo is 1.93 eV, which is more suitable than those for Bi_2_W(Mo)O_6_:Mo and Bi_2_W(W)O_6_ with ΔG_*OH_ of 1.41 eV and ΔG_*OH_ of 1.62 eV, respectively. Such a ΔG_*OH_ value (1.93 eV) between 1.6 and 2.4 eV is more favorable to H_2_O_2_ production (Figure c–f). DFT result suggests that W atoms in the Mo‐doped Bi_2_WO_6_ may be the active center, which is beneficial for the H_2_O_2_ production via 2e‐WOR. It was anticipated that the substitution of Mo for W in Bi_2_WO_6_ would cause a subtle alteration to the crystal structure and electron configuration, which would have a positive impact on the catalytic activity and charge transfer properties.

Xue et al. have reported the 2e‐WOR performance in acid media by the use of a C, N‐codoped TiO_2_ electrode. The electrode is made up of in situ‐produced, highly ordered TiO_2_ nanotubes doped with nitrogen (N) and carbon (C) on a Ti substrate.^[120]^ To investigate the doping effect, the untreated TiO_2_ (denoted untreated), air‐annealed TiO_2_ at 600 °C (denoted 600 A) and urea‐annealed TiO_2_ at different temperature (denoted 400 N, 500 N, 600 N, and 700 N, respectively) were prepared. Compared with the other catalysts, the treated 600 N exhibits the highest current density within a wide potential range (1.8–3.3 V vs Ag/AgCl) and the highest production rate. The highest H_2_O_2_ productivity for the 600 N can be obtained at 2.9 V vs. Ag/AgCl, reaching a cumulative H_2_O_2_ concentration of 0.85 μmol L^−1^. Moreover, the highest FE for H_2_O_2_ production on the 600 N was calculated to be about 13.0 % at 2.6 V vs. Ag/AgCl, along with a cumulative H_2_O_2_ concentration of 0.65 μmol L^−1^. The researchers found that the catalytic performance could be regenerated via re‐calcination strategy under urea conditions which provides a novel strategy to recycle the deactivated electrocatalyst. To explore the deactivation, various characterizations (such as EIS, SEM etc.) were applied to the 600 N before and after the stability test. They found that the change in electron transfer rate (Rct) is prominent for the 600 N sample before/after 6 h electrolysis while other characterizations reveal no obvious changes, indicating that the deactivation of the 600 N sample during long‐term electrolysis is primarily ascribed to the decrease in conductivity due to the oxidation of the deeply seated Ti current collector.

Sun et al. controllably introduced Ru single atoms into rutile TiO_2_ (Ru_x_Ti_1–x_O_2_), which effectively improved the activity of 2e‐WOR production of H_2_O_2_.^[114]^ The Ru_0.08_Ti_0.92_O_2_ catalyst demonstrate a high H_2_O_2_ Faraday efficiency up to 62.8 %, with an H_2_O_2_ yield of 24.2 μmol min^−1^ cm^−2^ (at 3.1 V vs. RHE for 10 min, current density up to 120 mA cm^−2^). At 3.1 V vs. RHE, the Faraday efficiency of H_2_O_2_ increased from 11.7 % (TiO_2_) to 41.0 % (Ru_0.08_Ti_0.92_O_2_) when Ru (3–4 %) was introduced into TiO_2_, suggesting that the Ru^3+^ site in Ru‐O−Ti can effectively convert the 4e^−^WOR pathway to the 2e‐WOR pathway, and thus effectively improving the H_2_O_2_ selectivity (Figure 7g). Experimental results and theoretical calculations show the Ru substitution of Ti centers remains periodically oriented, leading to the electrochemical and structural stability of the catalysts. In addition, the value of ΔG_*OH_ on the Ru atoms is close to 1.76 eV, which is the ideal value for H_2_O_2_ production via 2e‐WOR, and is conducive to achieving a highly selective production of H_2_O_2_ (Figure 7h–i). The Ru_x_Ti_1–x_O_2_ catalysts exhibit high H_2_O_2_ yields and Faraday efficiencies over a wide range of doping concentrations and potential windows, showing great potential for industrial production and practical operation.

In conclusion, doping engineering, which introduce impurity atoms into a material to change its properties‐is a crucial tactic for altering electronic structures of catalytic sites and boosting the activity of pristine catalysts, hence increasing the 2e‐WOR activity.

### 4.3 Facet Engineering of Metal Oxides

2e‐WOR usually take place on the surface of the catalyst, the catalyst′s electrochemical activity is closely associated with the active sites that are exposed in its various facets. To maximize catalytic reaction performance and clarify reaction mechanisms, it is essential to comprehend variations in the surface energy of crystal facets that affect intermediates adsorption. By controlling the nucleation and growth of the crystal, the facet engineering has been regarded as a useful strategy to realize controlled exposure of the crystal surface.^[132]^

The ZnO catalysts exposed with (1010) and (0001) crystal facets were prepared by Nørskov and Zheng′s group to evaluate the facet effect on the catalytic efficiency of 2e‐WOR.[^4^, ^117^] The as‐prepared (1010) ZnO features the morphology of nanorods and the (0001) ZnO is in the shape of nanoparticle.

**Figure 8:**
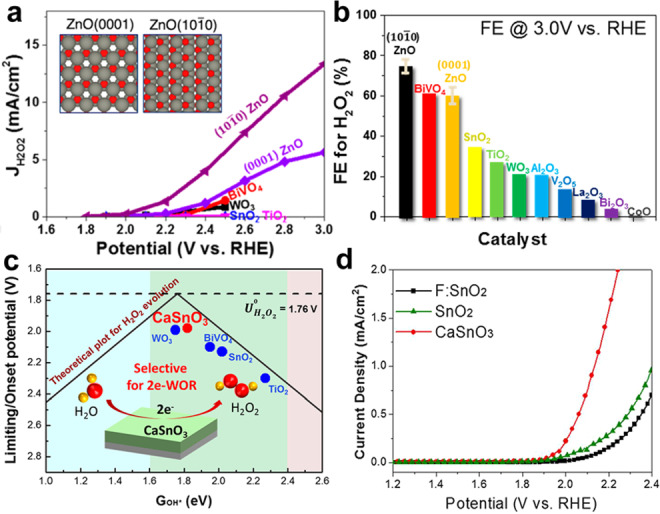
(a) Comparison of the catalytic activity by current density J−V curves for two different crystal facets of ZnO during 2e‐WOR. (b) Comparison of FE at 3.0 V vs RHE for (1010) and (0001) ZnO with previously reported studies. Reprinted (adapted) with permission from ref. ^[4]^ Copyright 2019 American Chemical Society. (c) CaSnO_3_ as the effective and selective electrocatalyst for the 2e‐WOR of H_2_O_2_ production. (d) J–V curve of FTO, SnO_2_, and CaSnO_3_ as the electrocatalyst for 2e‐WOR. Reprinted (adapted) with permission from ref. [52] Copyright 2019 American Chemical Society.

DFT calculations predict that (1010) ZnO is more suitable for H_2_O_2_ production via 2e‐WOR. The free energy diagram illustrates that the realization of the largest thermodynamic driving force toward H_2_O_2_ is based on two factors: first, the H_2_O_2_ in solution is more stable than adsorbed O* (ΔG_H2O2 [3.5 eV]_<ΔG_O*_); secondly, the adsorbed *OH is more stable than OH radicals in solution (ΔG_*OH_<ΔG_OH⋅_). According to the calculated results, ZnO (1010) and ZnO (0001) are both favorable to H_2_O_2_ production, particularly, the ZnO(1010) locates at the top of the activity volcano, indicating the terminating facet can influence the electrocatalytic performance despite the same crystal orientation and thermodynamically favorable. As shown in Figure 8a–b, the experimental results confirm that (1010) ZnO delivers higher performance than (0001) ZnO within a wide potential range of 2.0–3.2 V versus RHE, reaching the highest FE of 81 % at 2.6 V versus RHE and an overpotential of 40 mV at 0.1 mA cm^−2^.^[4]^ Compared with other transition metal oxides, such as SnO_2_, TiO_2_, WO_3_, and the typical BiVO_4_, the (1010) ZnO shows higher FE for H_2_O_2_ production at 3.0 V vs. RHE.

Zhang et al. synthesized three BiVO_4_ electrodes using the hydrothermal approach; these electrodes possess cuboid, truncated pyramidal, and quasi‐pyramidal morphologies, respectively.^[117]^ Note the synthesized BiVO_4_ all demonstrate a monoclinic structure with an intense [040] peak. As a result, they were all preferentially orientated along the same [010] direction. The 2e‐WOR current density of BiVO_4_ electrode showed a positive correlation with the [010] facet ratio. In the potential range of 0.6–1.8 V vs. RHE, the average FE (H_2_O_2_) for the [010] facet terminated samples reached as high as 70 %. It is expected that the surface states of the various facets will cause quasi‐Fermi level changes and energy band bending, which will regulate the charge transport rate and oxidizing capacity of the holes and change the reaction pathways.

Perovskite oxides have garnered a lot of attention for a variety of uses, such as electrochemical catalysts. Commonly, more than 90 % of the metallic elements from the periodic table replace the A and B site cations in the ABO_3_ structure. The adaptable structure offers sample possibilities for adjusting their electronic structure to enhance their selectivity for H_2_O_2_ production. CaSnO_3_ was suggested by Park et al. as a potential electrocatalyst for 2e‐WOR.^[98]^ Since the active sites on each facet of CaSnO_3_ varies and results different oxygen binding energy, the selectivity of different facets are investigated. The CaSnO_3_ [001] was predicted to be the best among the three facets (001, 010 and 100) because its ΔG_HO*_ is closest to the volcano′s peak (1.76 eV). Experimental results confirm that CaSnO_3_ [001] yields a greater %FE than other facets.

**Figure 9:**
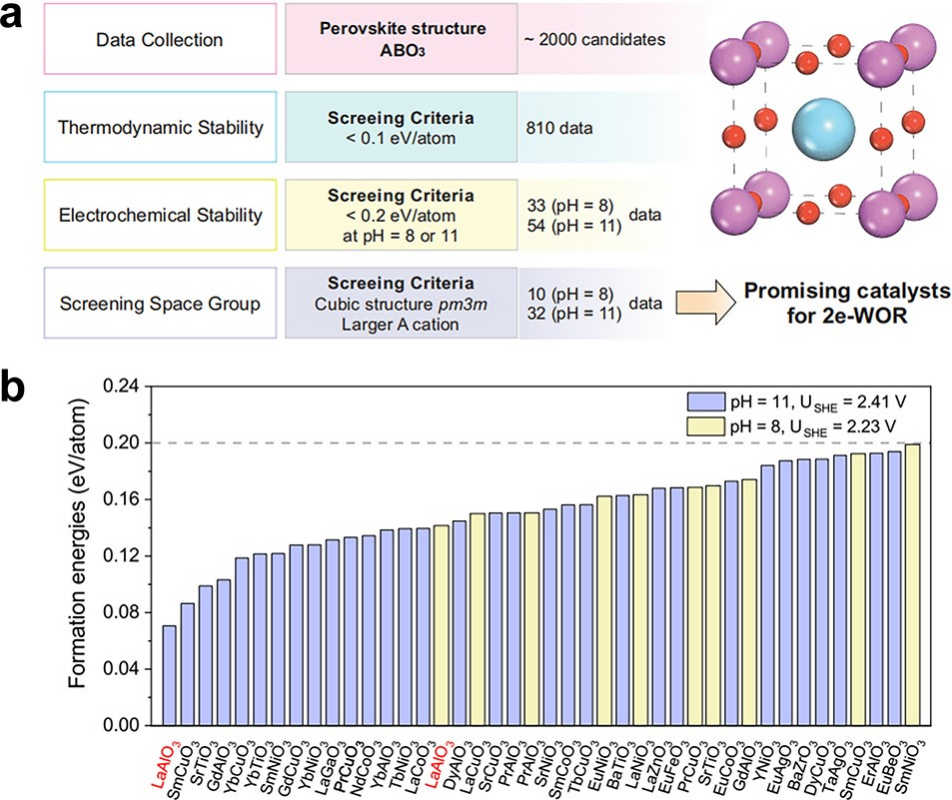
(A) Filtering stable ABO_3_ perovskite structures based on three screening criteria including decomposition energy <0.1 eV and electro‐chemical stability under pH=8 and 11. (B) Computational screening to discover the most stable perovskite oxide under different pH conditions. Reprinted (adapted) with permission from ref. [7] Copyright 2022 Springer Nature.

Furthermore, In 2022, Zheng et al. conducted an interesting research by integrating experimental methods with DFT calculations to examine the optimal ABO_3_‐structured electrode for on‐site anodic H_2_O_2_ electrosynthesis.^[7]^ The calculated limiting potentials indicate that LaAlO_3_ exhibits the highest stability, activity, and selectivity for 2e‐WOR among the various metal oxides (Figure 9a). Experimental confirmation demonstrated that the synthesized LaAlO_3_ has a cubic crystal structure with major crystal facets (100) and (110). LaAlO_3_ has good 2e‐WOR activity (the current density can reach 10 mA cm^−2^ at 510 mV overpotential) (Figure 9b), good selectivity (87 % peak FE at 3.34 V) and stability (only 3 % FE decrease after 3 h) in 4 M K_2_CO_3_/KHCO_3_, which is lower than the overpotential of numerous reported metal oxide catalysts. LaAlO_3_ is a highly effective catalyst for producing anodic H_2_O_2_.

### 4.4 Interface Engineering of Metal Oxides

Besides the abovementioned strategies, interface engineering is also powerful to regulate the catalytic performance via introducing another component/phase to form a heterojunction interface. The heterojunction interface can generate a built‐in electric field which can not only influence the charge transfer, but also activate the adsorption of intermediates, and thus tailor the catalytic performance.^[134]^

Gong and Zhang et al. prepared Co_3_O_4_/TiO_2_ heterojunction by a facile dip‐coating.^[47]^ The Co_3_O_4_ nanoparticles (≈10 nm) were successfully coated on the surface of TiO_2_ nanorods. Figure clearly exhibits that the coating of 0.25 wt % Co_3_O_4_ on TiO_2_ suppresses the production rate of O_2_. Further products analysis confirms that the suppressed OER is beneficial to 2e‐WOR for H_2_O_2_ generation. Compared with the pristine TiO_2_, the FE of H_2_O_2_ over 0.25 wt % Co_3_O_4_/TiO_2_ increases from 4.5 %–26.76 %. Photoluminescence study on OH⋅ radicals reveals that the accelerated 2e‐WOR is due to the promoted activation of water molecules. Also, this strategy can be extended to the WO_3_ substrate. For example, the resulting Co_3_O_4_/WO_3_ exhibits promoted photocurrent density within the range of 0.6–1.2 V versus RHE and reaches the current density of 0.86 mA cm^−2^ at 1.2 V vs. RHE. More importantly, the FE for H_2_O_2_ production can be observed from 6.2 % for WO_3_ to 12.2 % for Co_3_O_4_/TiO_2_.

At the same time, Park and Zhang prepared SnO_2_/BiVO_4_, and SnO_2–x_/BiVO_4_ heterojunctions via a two‐step electrodeposition followed by annealing at 450 °C under air and argon (Ar) conditions, respectively.^[139]^ The cross‐sectional SEM image reveals a total thickness of ≈800 nm for the SnO_2–x_/BiVO_4_ heterojunction. High resolution TEM image shows that a 6 nm thick SnO_2_ layer is covered on the surface of highly crystalline monoclinic BiVO_4_. The obvious interface between SnO_2–x_ and BiVO_4_ proves the successful formation of heterojunction. The corresponding element mapping images demonstrate the uniform distribution of Sn, Bi, and V elements over the SnO_2–x_/BiVO_4_ heterojunction. The authors found that either SnO_2_ or SnO_2–x_ overlayer on BiVO_4_ could suppress OER and H_2_O_2_ decomposition. The LSV curves clearly demonstrate that the current density of BiVO_4_ is reduced obviously under positive potentials after coating both SnO_2_ and SnO_2–x_ overlayer, indicative of the suppressed OER on BiVO_4_. The %FE of H_2_O_2_ increases from 50 %–88 % (SnO_2_) and 81 % (SnO_2–x_), respectively. To further examine the effect of SnO_2_ and SnO_2–x_ overlayer to inhibit the decomposition of generated H_2_O_2_, a RRDE technique was then employed. The ring currents of H_2_O_2_ decomposition on SnO_2_, and SnO_2–x_ are smaller than that on BiVO_4_ despite the same disk current, indicating the suppressed H_2_O_2_ decomposition. Moreover, under the photoelectrochemical conditions, coating SnO_2–x_ can further reduce the band bending of the BiVO_4_ to convert 2e^−^/4e^−^WOR to 1e^−^/2e^−^ reaction, such that a high FE of 86 % for H_2_O_2_ generation can be achieved. The catalytic activity and selectivity are directly affected by the interfacial structure, which is closely linked to the adsorption strength of the surface and influenced by its electronic structure.

Furthermore, researchers have created a few additional 2e‐WOR catalytic synthetic ideas in addition to the catalyst design methods already discussed. Hu et al. developed a two‐site highly selective ZnGa_2_O_4_ anode for the production of H_2_O_2_ by 2e‐WOR, which exhibited a high FE of 82 % at a low overpotential of 540 mV, as well as good stability.^[113]^ Based on experimental observations and theoretical calculations, H_2_O_2_ is formed on ZnGa_2_O_4_ via indirect and direct reaction pathways. More importantly, the indirect pathway mediated by HCO_3_^−^/HCO_4_^−^ is the key pathway for the production of H_2_O_2_. ZnGa_2_O_4_ facilitates the adsorption of HCO_3_^−^ on the double Ga−Ga sites. The conversion of *HCO_3_ to *HCO_4_ on ZnGa_2_O_4_ was confirmed by the energy changes in the in situ ATR‐FTIR spectra and DFT results. So, in catalytic research, controlling interfacial electronic effects and reaction pathways chemically to achieve the optimum adsorption strength of critical intermediates is a difficult task. Additionally, identifying important intermediates for complex reactions remains unclear. Hence, in order to understand the conformational relationships of complicated reactions and guide the design of catalytic materials for crucial reactions, it is essential to develop systematic and detailed ideas for exploring dynamic changes in interfaces.

**Figure 10:**
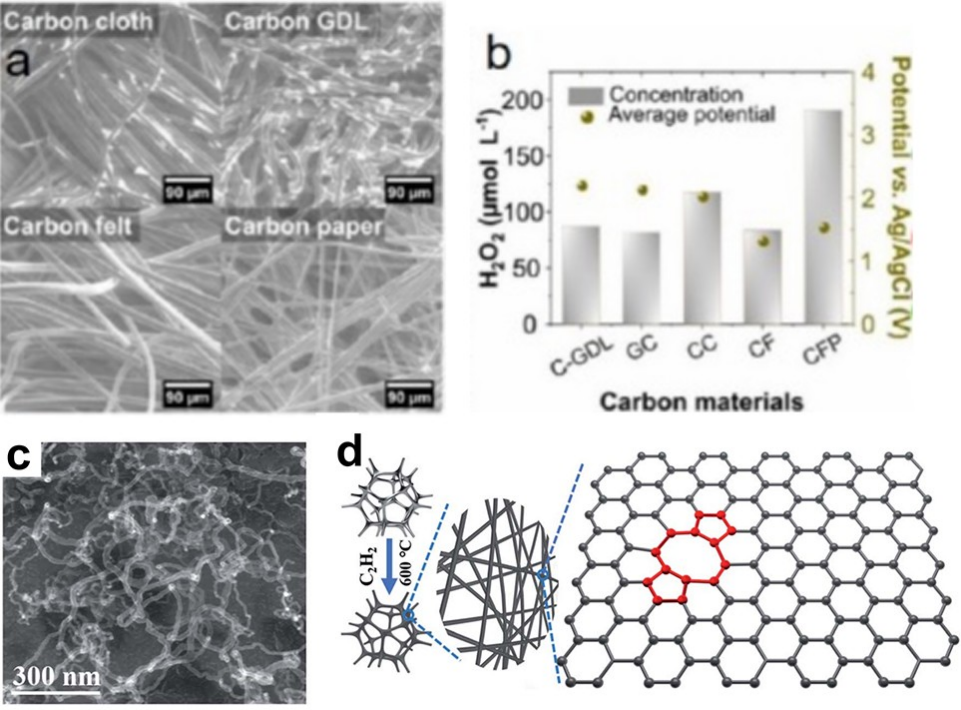
(a) SEM images of different carbon materials for anodic H_2_O_2_ production. (b) H_2_O_2_ concentration during anodic electrolysis using different carbon materials at 50 mA cm^−2^ for 10 min at room temperature. Reprinted (adapted) with permission from ref. [77] Copyright 2022 Elsevier. (c) SEM images of CNFs/NF. (d) Schematic of the formation process of CNFs/NF. Reprinted (adapted) with permission from ref. [75] Copyright 2021 Royal Society of Chemistry.

The carbon‐based electrodes’ design has received a lot of attention in the experimental and theoretical fields of H_2_O_2_ electrochemical synthesis in recent years.^[124]^ This shift is motivated by the limitations associated with high cost metals and limited availability. As a result, there has been a surge in carbon based materials as alternative, cost‐effective, and abundant electrocatalysts.^[140]^ The reported carbon based materials include BDD, CFP/PTFE, and defected carbon fiber, etc; compared with metal oxide, the carbon based materials usually demonstrate small overpotential, large current density, high production rate. The explanation might be due to the its excellent conductivity, high surface area, porous structure, versail active site, as well as adjustable interface (Figure 10a–b). However, carbon based materials often display low FE compared to the metal oxide, which might be the competitive reaction on the active sites.^[107]^ Thus, the primary focus in the research and development of carbon‐based materials lies in modulating catalysts and interfacial three‐phase reactions to improve selectivity. Besides, the durability of carbon based materials should be considered for the high working potential. The 2e‐WOR performance can be enhanced by adjusting the surface or bulk composition and modifying the structure of carbon based materials. thereby influencing the interfacial behavior between gas, liquid, and solid phases.

### 4.5 Defect Engineering of Carbon Fiber Materials

Liu et al. conducted an electrochemical synthesis of H_2_O_2_ using carbon fiber materials as anode. The process commenced with the utilization of a chemical vapor deposition technique on nickel foam to produce inherently defective carbon nanofibers (CNFs).^[122]^ The SEM image clearly illustrates the porous structure of the defect‐rich CNFs/NF electrocatalysts (Figure 10c). A current density of 72.6 mA cm^−2^ was attained at 2.9 V vs. RHE. DFT calculations revealed that the significant production of H_2_O_2_ can be attributed to the reduction in the absorption intensity of the OH* bond on ring defects of CNF/NF‐600 catalysts. A notable inhibition of the 4e‐WOR process was observed on this site (Figure 10d). Thus, the enhanced selectivity is primarily associated with the inherent defects. Subsequently, they fabricate a CNF/NF composite catalyst by the use of PTFE. The CNFs/NF@PTFE exhibited outstanding stability, maintaining approximately 48.1 % selectivity for H_2_O_2_ production even after 8 hours of continuous electrolysis at 2.8 V vs. RHE.

### 4.6 Doping Engineering of BDD

Among carbon‐based materials, Boron‐doped diamond (BDD) electrodes are widely recognized for their exceptional stability under high oxidized potential.^[141]^ BDDs with varying B/C composition ratiosalter the sp^2^/sp^3^ ratio, and elicit distinct electrochemical characteristics.^[142]^

In 2003, Michaud et al. documented the initial occurrence of H_2_O_2_ production via the process of water oxidation.^[145]^ The application of BDD electrodes for 2e‐WOR is related to several factors, including the boron doping concentration, the film thickness, and the crystal size of the diamond film. Ponce de Leon and colleagues conducted 2e‐WOR using a 12 um thickness of BDD film on Ti substrate.^[63]^ They investigated the electrochemical efficiency of anodic H_2_O_2_ production under alkaline conditions with 2.0 M KHCO_3_. At 3.47 V vs RHE, the maximum concentration and production rate of H_2_O_2_ attained were 29.0 mM and 19.7 μmol min^−1^ cm^−2^, respectively. The authors propose the existence of a specific potential range wherein the BDD exhibits a preference for H_2_O_2_ production over O_2_ evolution.

**Figure 11:**
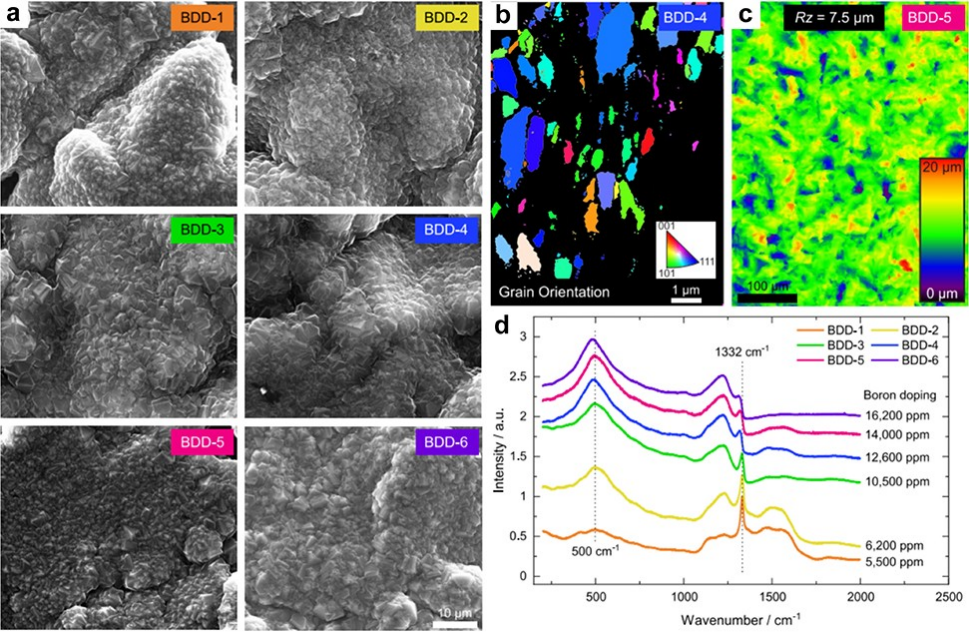
(a) SEM images of the six modulated BDD films. (b) EBSD analysis of BDD‐4. (c) LSM image of BDD‐5. (d) Raman spectra of the BDD coatings. Reprinted (adapted) with permission from ref. [27a] Copyright 2021 American Chemical Society.

An additional strategy to improve the electrochemical properties of BDD involves controlling the boron doping level. Mavrikis et al. have fabricated six distinct boron‐to‐carbon ratios of oxygen‐terminated BDD microfilms to evaluate its electrocatalytic performance in a hybrid carbonate/bicarbonate electrolyte(Figure 11a–c).^[63]^ A boron doping level of 12600 ppm and a layer thickness of 2 μm achieved the best 2e‐WOR. The performance, which FE is 87 %, the maximum H_2_O_2_ concentration of 29 mM, and a production rate of 76.4 μmol cm^−2^ min^−1^. This performance was sustained for 10 hours in a durability test. The Raman spectra of six BDD films with varying doping levels demonstrated an inverse correlation between the boron concentration in the layer and the sp^3^ diamond signal at 1332 cm^−1^ (Figure 11d). The bands of boron and its related compounds exhibited a progressive increase with increasing B doping level.^[126]^ In addition to substituting boron with alternative dopant atoms, manipulating distinct diamond sp^3^/sp^2^ ratios, creating novel BDD film structures, and engineering interfaces could be pivotal in enhancing their capacity for 2e‐WOR. A potential strategy for advancing BDD electrodes involves their combination with cocatalysts. This approach would prevent H_2_O_2_ breakdown and reduce the overpotential of the applied electrode. The high currents and superior electrochemical stability of BDD electrodes render them promising catalysts.

### 4.7 Interface Engineering of CFP

Pangotra et al. conducted a comparative study involving various unmodified commercial carbon materials, including carbon cloth, carbon fiber paper (CFP), carbon felt (CF), glassy carbon (GC), and carbon gas diffusion layer (CGDL), as anodic electrode materials for electrocatalytic H_2_O_2_ production in alkaline solutions(Figure 10a).^[124]^ Among these materials, CFP demonstrated the highest H_2_O_2_ concentration, reaching 270 μmol L^−1^ at 2.0 V vs. Ag/AgCl (Figure 10b). The explanation might be that the CFPs have high porosity, a wide surface area, and superior hydrophobicity, permeability, and porosity properties. Consequently, the researchers suggested that 3D structured carbon materials exhibit greater activity compared to 2D carbon electrode structures. However, due to the competing reaction of H_2_O_2_ decomposition, the low Faradaic efficiency (FE) and production rate of H_2_O_2_ necessitate structural engineering and surface modification of commercial carbon material to enhance selectivity toward H_2_O_2_.

**Figure 12:**
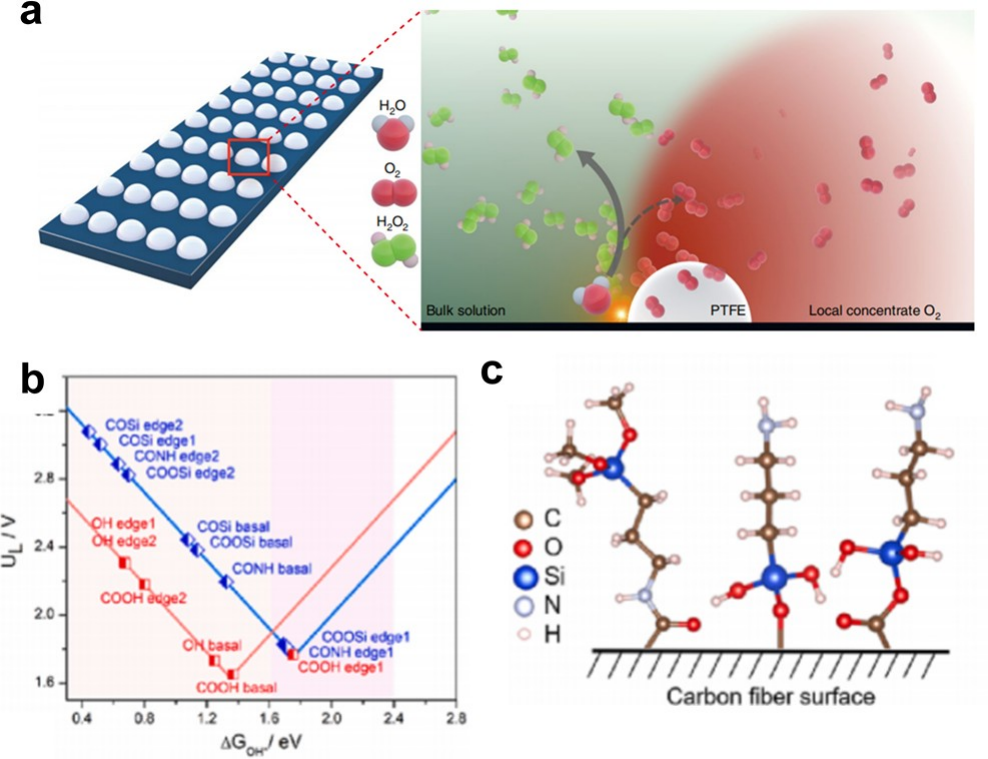
(a) A schematic showing the assumed possible reaction pathway tuning by local concentrate product. Reprinted (adapted) with permission from ref. [6] Copyright 2020 Springer Nature. (b) Water oxidation activity map of nine configurations of N‐CFP. The red and blue lines represent OER and 2e‐WOR activity, respectively. (c) Three ways for APTMS molecule to link with CFP. Reprinted (adapted) with permission from ref. [8] Copyright 2023 Elsevier.

Xia et al. introduced an interfacial engineering utilizing carbon fiber paper (CFP) coated with PTFE nanoparticles via spray application to facilitate 2e‐WOR (Figure 12a).^[6]^ The crucial aspect of regulating the WOR pathway relies on the interaction between the catalytic surface and the O intermediates (*O, *OH, and *OOH). The oxygen generated on‐site is gathered using PTFE “islands”. Subsequently, the confined O_2_ interacts with O* present on the electrode surface, thereby altering or the quantity of O* that surrounds the catalytic sites. The presence of locally trapped O_2_ gas modifies the binding energy of OH*. Consequently, the capture of O_2_ on the surface of the CFP anode is anticipated to reduce the binding energy of HO* due to the absence of hydrogen bonding networks. This would result in a reduction in solvation and cause a shift in the ΔG_HO*_ of the active site towards the upper end of the active volcano. Moreover, decreasing the water concentration in the vicinity of the electrode surface will restrict the oxidation of the catalyst surfaces. In a certain range of PTFE coating amount, with the increase of PTFE on the CFP electrodes, there was a steady decrease in the total current density, however, the selectivity for H_2_O_2_ increased. The Faradaic efficiency for H_2_O_2_ sixfold to 66 % compared to CFP, while the rate of H_2_O_2_ production to be 23.4 μmol min^−1^ cm^−2^.

The development of effective carbon‐based catalyst materials requires addressing not only the interaction of OH* free‐energy but also understanding the impact of the interfacial microenvironment on the catalytic reaction. This is conducive to a deeper exploration of the complex mechanism of H_2_O_2_ generation. For instance, Wang et al. developed a highly active 2e‐WOR electrocatalyst by surface modification of carbon fiber paper (CFP) using self‐assembled membranes (SAMs).^[8]^ The introduction of SAMs notably enhanced the 2e‐WOR performance of CFP. Electrochemical analysis revealed that the H_2_O_2_ generation rate of SAMs‐modified CFP reached 79.8 μmol min^−1^ cm^−2^ at a low potential of 2.1 V (vs. RHE), with an H_2_O_2_ selectivity of 82.5 %. Density functional theory (DFT) calculations revealed that SAMs modification could adjust the *OH binding energy of the CFP surface active site, while reducing the side reaction of the active site. This adjustment significantly increases the rate and selectivity of H_2_O_2_ generation (Figure 12b). Particularly, the authors suggest that the electrons directly transfer through the carbon atoms adjacent to the O=C−M bond during the 2e‐WOR process rather than via electron tunneling mechanisms (Figure 12c). Therefore, the 2e‐WOR performance remains unaffected by the functional tailing groups of the SAMs.

Very recently, Pascal Van Der Voort et al. presented a tailored covalent triazine network with strong oxidizing properties, serving as a proof‐of‐concept for a metal‐free organic network electrocatalyst for 2e‐WOR.^[146]^ The researchers opted for hexaazatriphenylene (HAT) due to its superb oxidative characteristics as the monomer for CTF synthesis. They then catalyzed the cyclization of the nitrile functional group in the HAT‐CN6 monomer to triazine using standard triflic acid, resulting in the extended organonetwork: HAT‐CTF. The successful synthesis of HAT‐CTF was validated through XRD, TEM, and FTIR analyses, with further confirmation provided by CP‐NMR. Additionally, XPS analysis corroborated the structure of HAT‐CTF. The electron‐by‐electron transfer process occurring during the 2e‐WOR process on the HAT‐CTF surface was validated through analysis of the Tafel plots derived from the polarization curves and the Nyquist plots obtained from the EIS analyses. This process involves the initial transfer of electrons between the catalyst surface and water to form the CTF‐OH* intermediate, followed by another set of electrons transferring to two such intermediates, resulting in consecutive coupling and H_2_O_2_ production. Further work for the detailed reaction mechanism and structural changes during catalysis and regeneration was provided by the analysis of impedance spectra and DFT calculations. Several results affirmed the dependence of HAT‐CTF on electrolyte and pH, with the maximum H_2_O_2_ yield observed at pH 12. Under optimal conditions (1 M K_2_CO_3_, 3.0 V vs. RHE), the CTF demonstrated a %FE of 89.9 and an H_2_O_2_ production rate of 1428 μmol/h/cm^2^ over a reaction time of 2 hours, comparable to state‐of‐the‐art electrocatalysts.

## 5 Three‐Phase Interface Engineering

In addition to the electrocatalyst material, factors such as the electrolyte, temperature, H_2_O_2_ concentration, applied external bias(voltage/current), and impurities can affect the catalytic performance for 2e‐WOR. This section discusses these important factors related to 2e‐WOR interfacial process, such as the catalyst‐electrolyte and product‐electrolyte interfaces, which plays a key role in optimizing the chemical environment for local H_2_O_2_ evolution, overcoming mass transport limitations during H_2_O_2_ synthesis, and improving the overall H_2_O_2_ evolution rate. In contrast to molecular‐scale tuning for the catalyst, interfacial engineering includes surface modification, complexation of electrolytes on surfaces, and local environment pH manipulation. The theoretical simulations (including Molecular Dynamics (MD)/Ab Initio Molecular Dynamics (AIMD), Density Functional Theory (DFT)) can successfully mimic the complex interfacial environment with the surface structure of electrode, which help to comprehensively understand the catalytic system and optimize the reaction.

### 5.1 Effect of Local Protonation Environment

Because the 2e‐WOR processes involve proton transfer, the H_2_O_2_ production is influenced by the local protonation environment. The selectivity of the WOR process is mostly dependent on the protonation of OOH* intermediates. O_2_ is produced when OOH* is deprotonated, while H_2_O_2_ is produced when OOH* is protonated. The protonation or deprotonation process can be regulated by the local protonation environment.^[147]^

Varying the point of zero charge (PZC) or zeta potential of electrode surfaces can create a favorable protonation environment. It is reported that the thermodynamic reaction barriers for the 2e‐WOR follow the sequence of WO_3_ (with PZC 0.5–1.5)<BiVO_4_ (with PZC 2.5–3.5)<TiO_2_ (with PZC 4.7–6.1), which is consistent with their PZC values.[^66^, ^148^] This is because surfaces with low PZCs (<7) are acidic in water, which provides a protonation environment for the H_2_O_2_ evolution.^[149]^ Based on the same principle, catalysts also exhibit high selectivity for H_2_O_2_ when the surface has a positive zeta potential. Therefore, using either acidic reaction media or catalysts with low PZCs or positive zeta potentials can optimize the binding energy of OOH*, thus enhancing the selectivity for H_2_O_2_.^[150]^

### 5.2 Effect of Interface Microenvironment

The electrolyte not only acts as an ionic conductor but participate in the production and stabilization of H_2_O_2_. The HCO_3_^−^ or CO_3_^2−^ based electrolytes demonstrate superior H_2_O_2_ production activities compared to other commonly used electrolytes. In these electrolytes, unstable peroxo species, can oxidize the reactant (H_2_O) to the product (H_2_O_2_) via an indirect two‐step reaction, thereby facilitating the two‐electron conversion from H_2_O to H_2_O_2_. Bicarbonate‐mediated H_2_O can increase the synthesis of H_2_O_2_ by forming HCO_4_^−^ intermediates in solid‐liquid interface. It has been demonstrated that the yield of H_2_O_2_ increases as the concentration of HCO_3_^−^ increases from 0–2 M, regardless of applied potentials.^[2]^

For example, Wang et al. reported that fluorine‐doped tin oxide electrodes (FTO) in carbonate solutions could produce H_2_O_2_ selectivity of up to 87 % and H_2_O_2_ currents of up to 1.3 A cm^−2^. The water oxidation reaction′s route can be modulated from 4e^−^WOR to 2e‐WOR by this CO_2_/carbonate‐mediated method. Electron paramagnetic resonance and isotope labeling investigations have demonstrated that carbonate facilitates the 2e‐WOR pathway to generate H_2_O_2_ by producing carbonate radical intermediates and percarbonate intermediates. As a media for “electron shuttling,” the redox‐responsive mediators are crucial in improving the selectivity of electrocatalysis.

In another study, Hu et al. developed the two‐site highly selective ZnGa_2_O_4_ anode for the production of H_2_O_2_ by 2e‐WOR, which via indirect and direct reaction pathways. The indirect pathway mediated by HCO_3_^−^/HCO_4_^−^ is the key pathway for the production of H_2_O_2_. ZnGa_2_O_4_ facilitates the adsorption of HCO_3_^−^ on the double Ga−Ga sites. The conversion of *HCO_3_ to *HCO_4_ on ZnGa_2_O_4_ was confirmed by the energy changes in the in situ ATR‐FTIR spectra and DFT results. In particular, the adsorbed *HCO_4_ was found to decompose into *CO_2_ and *OOH, the latter being converted to H_2_O_2_, and the peroxygen bonds in H_2_O_2_ and HCO_4_^−^ are stabilized on the surface of ZnGa_2_O_4_ both with and without the HCO_3_^−^ anion.

Since the formation of stable complexes could indirectly affect the dynamics and selectivity of the reaction, it is important to design the complexation of the electrolyte with the key intermediates/products during the 2e‐WOR process.

### 5.3 Facilitating the 2e‐WOR Pathway

Since the O_2_ inevitably involve in the 2e‐WOR for the competent 4e‐WOR pathway, the three‐phase interfacial reactions (TPIR) play a key role because it represents the junction where the gas, liquid, and solid phases converge. It also affects electron/ion transport and catalysis at the electrode surface. The construction of micro‐nanostructures on the catalyst surface can effectively promote the release of bubbles generated during the 2e‐WOR catalytic process, preventing them from obscuring the catalytic active sites. Additionally, these structures can regulate the hydrophilicity of the catalytic material and enhance electrolyte penetration, which facilitates mass transfer between the catalyst and the electrolyte. This, in turn, leads to improved catalytic activity. The TPIR can be effectively regulated by modulating the active sites on the catalyst surface, or tuning the hydrophilicity or hydrophobicity, or introducing electron transfer mediators, or optimizing the morphology and structure of the electrode. The optimization of the TPIR help to improve the catalytic activity, selectivity, and overall reaction efficiency.

**Figure 13:**
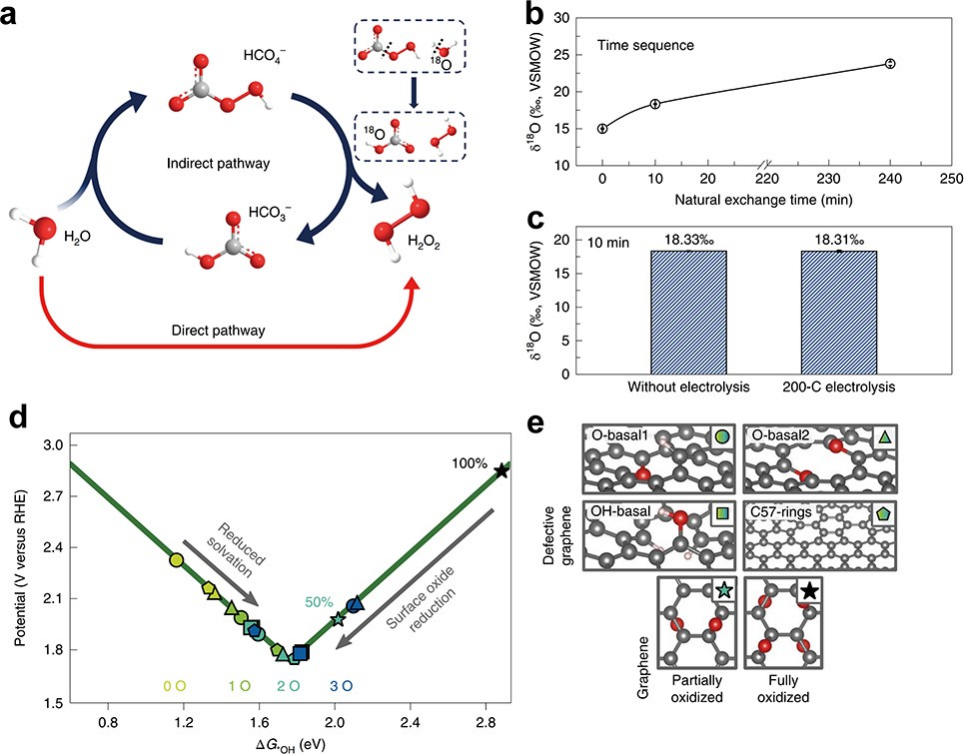
(a) Two different reaction pathways for catalytic two‐electron water oxidation. (b) 18O isotope abundance in quickly dried (within 2 min, Methods) 1.0 M Na_2_CO_3_ electrolyte with varied isotope exchange time between Na_2_CO_3_ solute and deionized water. (c) 18O isotope abundance in quickly dried 1.0 M Na_2_CO_3_ electrolyte with or without applied potential (2.4 V versus RHE). (d) The data points depict *OH binding energies on defected graphene sheets as well as partially (50 %) and fully oxidized graphene sheets (stars) (without correction for solvation effects) and are schematically drawn. (e) In the case of the defected structures, four different typical defects were considered as well as different coverages of oxygen atoms in the vicinity of the active site indicated. Arrows indicate potential external driving forces. Reprinted (adapted) with permission from ref. [6] Copyright 2020 Springer Nature.

Xia et al. performed electrochemical experiments using PTFE‐CFP in a Na_2_CO_3_ electrolyte. The hydrophobic PTFE “islands” on CFP is believed to trap side product O_2_, which would impact on the electrode surface, thereby optimizing the intermediates’ binding strength towards H_2_O_2_ generation. Oxygen isotope analysis confirmed that H_2_O_2_ was oxidized directly on electrode (Figure 13a–c), rather than via the formation of electrolyte mediated intermediates. The optimization of the CFP surface also involves the absence of hydrogen bonding networks, which lowers solvation. The optimization of local environment leads the active site′s ΔG_OH*_ toward the peak of the activity volcano (Figure 13b–d). Reactions that favor the two‐electron route are produced by these results.

### 5.4 Effect of Mass Transportation

The stability of the H_2_O_2_ generated at the interface are important for the production and further use. If the produced H_2_O_2_ cannot be released from the surface of electrode rapidly, it might experience the degradation in various ways, including heterogeneous redox at electrode or homogeneous reactions in bulk solution.

The existing approaches are still insufficient for the quick transmission of products. Surfactants can help intermediates to be released from the catalyst surface.^[152]^ The kinetic for H_2_O_2_ production can be improved by the fast diffusion of the intermediates. Ion‐exchange membranes (IEMs) allow for selective permeability to charged ions while blocking ions of opposite charges.^[153]^ This promotes mass transfer through Coulombic interactions. If hydrogen peroxide cannot be delivered rapidly, it will degrade in various of ways, including heterogeneous redox at an electrode or homogeneous reactions in bulk solution. Alternatively, to prevent H_2_O_2_ from breakdown at the catalytic interface, it is advantageous to transfer H_2_O_2_ under flowing electrolyte conditions immediately after it is produced in the field. And covering the active site will inhibit further reactions. The refreshing impact of water flow on the active surface in flow cell design has been demonstrated to be effective. By optimizing the residence time of the electrolyte in contact with the electrode surface in flow mode, it is possible to minimize the breakdown of in situ H_2_O_2_.

According to a study by Luciana Vieira et al., anode electrolyte circulation can reduce performance degradation.^[154]^ To maximize the continuous synthesis of accumulated H_2_O_2_, it is recommended to use a single‐pass flow mode. This prevents the electrochemically generated H_2_O_2_ from cycling back. With this single‐pass configuration, current densities of up to 700 mA cm^−2^ can be achieved. The electrolyte′s transient reaction at the reaction interface has a significant impact on overall electrolytic performance. To ensure the electrode surface is fully exposed to the reacting substances in solution, the single flow cell system is used to provides a uniform distribution of flow rate. This reduces mass transfer polarization and enhances reaction efficiency. The constant flow of electrolyte over the catalytic surface effectively carries away produced intermediates from the active site, preventing their accumulation and reducing the mass transfer resistance while maintaining the activity of the catalytic interface. At the same time, introducing fresh reactants refreshes the active site, maintaining stable reaction progress. Increasing the flow velocity near the active site reduces the residence time per unit volume of electrolyte, thereby mitigating related side effects.

Therefore, a single flow cell design can integrate Faradaic efficiency (%FE) and yield, control mass transfer rates, and reaction conditions flexibly. It can quickly replenish the electrode surface reaction environment and provide new catalytic opportunities for active sites. This refreshing mechanism is expected to extend the catalyst and product lifespan, prevent active site deactivation, and facilitate the rapid translocation of H_2_O_2_.^[154]^ Connecting the anode and cathode in flow cell mode can lead to a diversified increase in production value and potentially eliminate the need for stabilizers, resulting in cost savings. The single flow cell method provides an innovative solution for synthesizing 2e‐WOR and enhances catalytic stability and efficiency.

### 5.5 Complementary Theoretical Analysis of TPIR

When an electrode contacts the electrolyte, the ions in the electrolyte create a charge distribution on the electrode surface, forming a charge cloud around the electrode. This distribution leads to the formation of two electric layers on the electrode′s surface, which are known as the double electric layer. The bilayer theory is a commonly used explanation for this phenomenon. It proposes the existence of two interleaved layers: an ′outer layer’ containing adsorbed ions or molecules on the electrode surface, and an ′inner layer’ with ions within the electrolyte solution. The presence of these two layers affects the adsorption, desorption, and charge transfer of the ions in the electrolyte on the electrode surface. The solvent effect is a factor that influences the formation and characteristics of the bilayer. The solvent molecules may be encircled or wrapped by ions or molecules that are deposited on the electrode surface, primarily due to electrostatic interactions between the solvent and the electrode surface. The solvent effect is primarily influenced by the local pH micro‐environment and changes in solvent composition. Acids and bases in the solvent buffer the local pH, which is essential for maintaining the activity and selectivity of the catalyst. Additionally, the type and composition of the solvent influence its interactions and structure, which modifies the solute′s solubility, stability, and rate of reaction.

Electrolyte has an impact on the adsorbed species’ capacity to form hydrogen bonds with water molecules. researchers often simulate a bilayer H_2_O molecular structure in the system, which provides a well‐defined structure to understand solvent effects. Song et al. used an F doped WO_3_ anode to produce H_2_O_2_.^[155]^ DFT results reveal that the first stage of 2e‐WOR on WO_3_ was more favorable by the addition of F, and then facilitate desorption of HO* and O−O*. Particularly, the MD analysis suggest that the F doped WO_3_ changes the structure of interfacial water molecules, impacting the electrocatalytic activities for 2e‐WOR. The selectivity of the 2e‐WOR was maximally boosted by 84 % after the doping with F. The doped F causes the WO_3_ surface to become less polar and increases the dipole moments of the water molecules interacting with the surface, directing them towards the WO_3_ surface. This method polarizes the solvent through electrostatic interactions with the charges of the solute molecules. The charge distribution of the water molecule in the electrolyte is altered by this polarization, resulting in a decrease in the system′s energy, reactions are more likely to occur.

## 6 Electrochemical Cell Design

**Figure 14:**
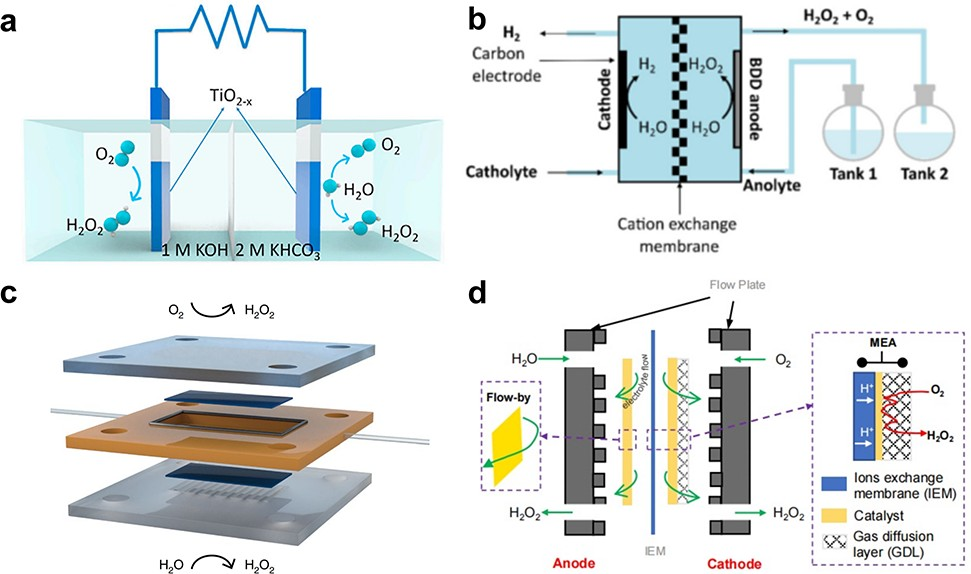
(a) Schematic illustration of the tandem cell using TiO_2–x_ as the bi‐functional electrode. value of TiO_2–x_ and pristine TiO_2_ via 2e‐WOR. Reprinted (adapted) with permission from ref. [59] Copyright 2021 Elsevier. (b) single pass electrochemical cell. Reprinted (adapted) with permission from ref. [97] Copyright 2022 Royal Society of Chemistry. (c) Schematic design of a coupled cell for H_2_O_2_ production. Reprinted (adapted) with permission from ref. [6] Copyright 2020 Springer Nature. (d) Flow‐by cell assembled with a gas diffusion electrode and a membrane separator. Reprinted (adapted) with permission from ref. [99] Copyright 2021 Elsevier.

At the device scale, our main focus is on the production of H_2_O_2_ at the anode from water oxidation, and the main differentiator across devices is the corresponding half reaction at the cathode. There exist two commonly used alternatives. The first cathode reaction option is the hydrogen evolution reaction (HER), which produces two useful molecules from a single electrochemical device: H_2_ (2H^+^+2e^−^→H_2_).Another choice is ORR, where O_2_ is reduced by 4e‐ORR and/or 2e‐ORR to H_2_O_2_. It is preferred to use 2e‐ORR to increase H_2_O_2_ generation. The creation of well‐designed electrochemical reactors and devices is essential to achieving high‐yield and practical H_2_O_2_ electrosynthesis. Single‐channel flow cells are necessary for experimental applications to avoid the disproportionation/decomposition of H_2_O_2_. The use of single pass flow cells should be considered for the mass transfer in coupled reaction devices during long term electrolysis. (Figure 14b).^[154]^

Dong et al. synthesized TiO_2–x_ via hydrogen plasma etching and presented an electrochemically driven bifunctional H_2_O_2_ production system (WOR/ORR cell) utilizing TiO_2–x_ catalysts (Figure 14a).^[105]^ the 2e‐WOR on the anode with 2 mol L^−1^ KHCO_3_ electrolyte and the 2e‐ORR on the cathode with 1 mol L^−1^ KOH electrolyte were coupled to create H_2_O_2,_ yielding a remarkable FE_H2O2_ of 134.2 % and a high H_2_O_2_ production of about 20 mmol L^−1^ h^−1^. Additionally, the electrochemical device may function in the presence of sunlight. In the absence of solar irradiation, the device operates as an electrolyzer. Under solar irradiation, the device functions as a PEC cell.

To achieve a desired rate and efficiency of H_2_O_2_ generation, Xia et al. presented a flow cell for H_2_O_2_ electrosynthesis with 2e‐WOR/2e‐ORR system (Figure 14c). At a tiny cell voltage of 1.7 V vs. RHE, the cell can obtain a high current of 120 mA cm^−2^ (WOR) and a whole‐cell FE_H2O2_ of 153 %. The practical application potential of this on‐site H_2_O_2_ flow generating technology were further demonstrated by its application in the degradation of organic contaminants in wastewater.^[6]^

It is possible to produce formate and H_2_O_2_ simultaneously by coupling cathodic CO_2_ reduction with anodic two‐electron water oxidation. This allows for the creation of important chemicals at both electrodes. Yu et al. reported a novel hybrid electrosynthesis strategy, using Zn‐doped SnO_2_ (Zn/SnO_2_) nanodots as a bifunctional electrocatalyst to achieve excellent stability for at least 60 hours at current densities of approximately 150 mA cm^−2^, and Faraday efficiencies (FEs) of 80.6 % and 92.2 % for the production of H_2_O_2_ and formate, respectively.^[157]^ The researchers discovered that Zn dopants improved *OH intermediate coupling and facilitate the production of H_2_O_2_, accelerating formate generation through *OCHO intermediate adsorption optimization. This was discovered through a combination of physicochemical characterization, including operationally attenuated total reflection Fourier transform infrared spectroscopy (ATR‐FTIR), isotope‐labeled mass spectrometry (MS)/1H NMR, quasi‐in‐situ electron paramagnetic resonance (EPR), and density‐functional theory (DFT) calculations.

In the MEA system, hydrogen peroxide molecules produced at the catalyst‐membrane interface need to be transported through the pores of the catalyst layer and the GDL, and the design of this electrolytic cell has been much investigated in other fields (Figure 14d).^[156]^ It is expected to be applied to the 2e‐WOR, but a suitable counter‐electrode reaction is sought.

Moreover, the flow cell and light field can be combined to increase efficiency. A light‐driven fuel cell with spontaneous H_2_O_2_ and energy generation has been designed by Shi et al.^[56]^ Both the cathode‐evolved ORR and the photoanode‐catalyzed WOR were two‐electron reactions that produced H_2_O_2_. Under light, the n‐type semiconductor BiVO_4_ thermodynamically permitted the formation of H_2_O_2_ through 2e‐WOR and, because of its 2.4 eV bandgap without additional bias. The above‐described studies provide new insights into both bifunctional catalyst design and coupled cathode reaction.15, ^[156]^

**Figure 15:**
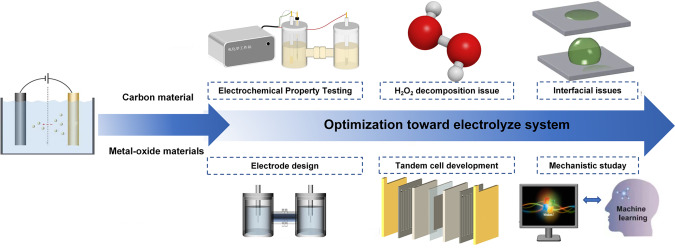
Promising directions for 2e‐WOR electrode and devices.

## 7 Summary

Electrochemical synthesis via 2e‐WOR is an emerging process of interest for in‐situ and sustainable production of H_2_O_2_. It provides an interesting pathway for distributed in‐situ H_2_O_2_ production under ambient conditions without gas phase reactants. We review the recent progress on 2e‐WOR, including fundamental principles, catalyst development, H_2_O_2_ detection and electrochemical system design. Theoretical calculations have played a major role in paving the way for mechanistic understanding of reaction and the design of novel catalysts. To understand the electrocatalyst and related reaction mechanism, we analyzed and discussed its synthesis strategy, components, structure, and related electrochemical properties, particularly, we focused on the selectivity, activity, and long‐term stability.

Designing efficient anode materials for the production of H_2_O_2_ via 2e‐WOR presents a challenge due to the need for the electrocatalyst to improve selectivity by inhibiting the thermodynamically favorable 4e‐WOR pathway. Numerous studies on WOR have been conducted using a combination of calculations and experiments. The selectivity of 2e‐WOR catalysts can be evaluated by considering the ΔG_OH*_ and ΔG_O*_ values on the material′s surface. The adsorption strength of OH* and O* on the surface is a determining factor for WOR selectivity. It is ideal for ΔG_OH*_ values to be between 1.6 and 2.38 eV, and ΔG_O*_ to be greater than 3.52 eV, as this range favors the formation of hydrogen peroxide. Theoretical calculations establish principles for predicting the activity and selectivity of 2e‐WOR electrocatalysts, particularly metal oxides and carbon‐based materials. The range of descriptors provided is a strong guide for screening efficient catalysts.

For industrial application, the 2e‐WOR catalyst durability is very important due to the high working potentials (∼3.0 V Vs. RHE), which often lead to catalytic activity degradation. Descriptor‐based analysis and new software tools can aid in predicting electrochemical stability of potential catalysts. To predict the electrochemical stability of catalysts, it is possible to narrow down many potential structures and create Pourbaix diagrams to assess electrochemical stability. The Pourbaix diagram is a useful thermodynamic tool.

Recently, a wide range of earth‐abundant materials, including carbon‐based materials, metal oxides, and metal complexes, are introduced as efficient electrocatalysts for anodic H_2_O_2_ production. Molecular porphyrins such as AlTMPyP and ZnTMPyP have been shown to produce H_2_O_2_ anodically at some of the lowest overpotentials reported to date. While metal oxides often show the impressive faradic efficiency of 2e‐WOR at low current densities, the carbon based materials often display high yield, particularly at a relatively low potential. Through engineering the electrode at molecular level, a high activity and selectivity can be obtained. It′s worth noting that the boron doped diamond demonstrates high durability in the alkaline media, suggesting potential industry application.

For 2e‐WOR, besides the properties of the catalysts themselves, by adjusting the chemical local microenvironment between the interface and the electrode surface, interactions between catalysts, reactants, intermediates, products, and electrolytes can be optimized. An important factor is the used electrolyte, which lead to the formation of different reaction intermediates such as HO*, HO−, (O−O)_2_−, HCO_4_− or peroxy complexes, and impact the activity and selectivity. Besides, the generation and desorption of specific products can be effectively promoted by changing roles of different intermediates in the active sites. Many studies have used carbonate or bicarbonate based compounds as electrolyte for the anion participate in the catalytic cycle through the formation of reaction intermediates. It has been postulated that the HCO_3_^−^ anion can contribute to enhanced H_2_O_2_ electrosynthesis by actively participating in the catalytic cycle through the formation of reaction intermediates and by stabilizing the generated H_2_O_2_ through the formation of peroxy adducts or complexes (such as hydrogen peroxocarbonate) that do not undergo further oxidation. So, highly efficient anodic H_2_O_2_ generation depends on synergistic effect between catalyst and electrolyte.

This review also summarizes the significance of interfacial engineering and the need to properly consider interfacial solvation effects in 2e‐WOR electrocatalytic calculations is also discussed. The study of interfacial reactions in electrocatalytic reactions is crucial for a deeper understanding and optimization of electrochemical processes. The charge or electric field distribution is non‐uniform in reality due to surface roughness. Molecular surface modifications can enhance and modulate catalyst performance. Some molecules have dielectric effects that can alter the interfacial electric field and the Facilitate 2e‐WOR reaction path. And certain molecules can change the structure of the double electric layer and affect the electric field distribution, thus altering the reaction process. For example, after modifying the surface of PTFE, the confined oxygen present modulates the water oxidation reaction path and enhances selectivity. And doping F can effectively deflect the angle of the interface between the first layer of molecular water and the reaction interface, thus regulating the distance between them. The rate of interfacial reactions can then be increased by adjusting the hydration valence or charge transfer resistance of the electrode surface. In conclusion, comprehensive modification of the catalysts (material structure or surface hydrophobicity/hydrophilicity) and changes in the interfacial microenvironment (electrochemical potential, local pH distribution or product concentration) affect the binding energy of the key 2e‐WOR intermediate, as well as the transport and stability of H_2_O_2_, and influence the dynamics, thermodynamics and reaction mechanism of the electrocatalytic reaction. These studies offer a strong theoretical foundation and experimental evidence for the advancement of interfacial engineering and related fields.

In this review, we have summarized some strategies for system engineering. As a whole electrochemical cell, the choice of cathodic coupling reaction is important. Generally, the HER (Hydrogen evolution reaction) is cathode reaction, the HER/2e‐WOR is commonly observed coupling reaction. To improve the capacity of H_2_O_2_ generation system, a 2e‐ORR/2e‐WOR coupled reaction is introduced, Improved energy utilization and more efficient H_2_O_2_ production while dual‐channel generation of the target product. A recent CO_2_RR/2‐WOR coupling reaction simultaneously obtain formic acid and H_2_O_2_, suggesting a great potential for cathodic coupling reaction.^[157]^ By optimizing the reaction conditions and catalysts design, such systems possess high conversion efficiency for electrochemically driven production of valuable chemicals. PEC (Photo electro chemical) H_2_O_2_ production is a sustainable and low energy consumption approach. Electrocatalysts for cathodic H_2_O_2_ production are also suitable for the PEC process.^[158]^ PEC focuses on solar energy conversion and light‐driven chemical reactions through the interaction of light‐absorbing materials with photogenerated charges. Although it is different from electrocatalysis in terms of research objects and mechanisms, they may have some cross‐applications in the field of solar energy conversion and renewable energy, such as combining photoabsorption and electrocatalysis and achieving higher production efficiency. In addition, given the convenience and practicality of solid electrolytes, the use of a porous solid electrolyte (PSE) layer instead of the liquid electrolyte is an effective approach, as demonstrated by Xia et al.^[106]^

## 8 Outlook

The majority of 2e‐WOR research to date has been on the basic understanding of electrocatalysts. Numerous factors influence the H_2_O_2_ accumulation and the catalytic performance of 2e‐WOR electrocatalysts, as shown in Figure 15. Since many of these important factors and their effects on the reaction mechanisms have not been thoroughly investigated, we believe that future research in these areas is necessary:

1. It is necessary to develop catalysts that are both active and selective, not only favouring the two‐electron pathway rather than the four‐electron pathway, but also inhibiting the decomposition and further transformation of H_2_O_2_, by adjusting the type and location of doped elements and activating the path of the 2e‐WOR. Therefore, judicious calculation can speed up the development of catalysts and reduce the associated costs in searching for suitable catalyst materials. The next generation of materials will most likely emerge from a combined computational and experimental approach, where parametric simulations will narrow a large pool of materials down to a shortlist of likely catalyst candidates based on their surface binding energies, which can then be experimentally tested for performance.
2. The electrochemical synthesis of H_2_O_2_ via 2e‐WOR involves higher oxidation potentials, it can lead to degradation of metal and metal oxide catalysts. Further research should focus more on materials that exhibit prolonged stability under 2e‐WOR conditions. With recent developments in DFT, Pourbiax diagrams can be rapidly constructed and used to assess electrochemical stability.
3. Compared to the more mature oxide materials, there is still a lot of work to be done in the future as low cost and easily modified carbon‐based materials. Sp_3_ carbon active sites have been shown to increase productivity. In addition to the researched material, diamond‐like carbon (DLC) or hydrogen‐carbonized hierarchically porous carbon (HPC), may give an interesting low‐cost option. Modified graphene or graphene oxide electrodes may also be suitable carbon‐based alternatives for the 2e‐WOR. In the future, these materials can be tailored with nanoparticle loading, elemental doping or surface structure tuning to withstand oxidation. These are promising approaches to generate both charged and high spin density sites.
4. We recommend that long‐term stability tests for catalysts and devices should be performed for as long as 100–1000 h whenever possible to understand the degradation mechanism and develop benchmarking protocols. Besides, it is urgent to establish the standard H_2_O_2_ quantification approach to ensure the fairness of catalytic activity. Quantification of H_2_O_2_ concentration is a prerequisite for the detection of 2e‐WOR. In‐situ quantify approach can help to eliminate the huge error of H_2_O_2_ concentration.
5. The majority of theoretical studies on 2e‐WOR have so far focused on electrocatalysts, neglecting that the study of interfacial reactions in electrocatalytic reactions. Due to the complexity of electrochemical three‐phase interfaces, electrochemical interface studies of 2e‐WOR face many challenges. The main one is the lag in the theory of interfacial structure, especially the lack of in‐depth study on the structure of atypical electrochemical three‐phase interfaces; secondly, the separation of electric and non‐electric field effects, which requires further study of the correlations and mechanisms; and finally, the realization of the precise localization of the adsorbed ions, which is currently an average value of the adsorption of ions on the surface of the catalysts, and most of them are dependent on the theoretical simulations. In the future, the combination of DFT and MD/AIMD will be a powerful theoretical tool to elucidate the mechanism of the actual reaction at the three‐phase interface, and the implementation of ML (machine learning) will be a key way to accelerate the screening of highly stable and selective 2e‐WOR catalysts. The synergy of atomic modelling, machine learning and multiscale modelling facilitates the screening and discovery of active sites, combined theoretical modelling and experimental studies of the energetics, kinetics, charges and molecular binding strength of modified surfaces should accelerate the development of catalysts, electrodes and devices.
6. In addition to the active site itself, the local reaction environment should also be evaluated. Surface and interfacial modifications offer prospects for tailoring the reaction pathway and influencing the protonation step. The type of electrolyte solution, pH value, penetration/wetting of water, short‐range mass of water near the active site and proton transfer have great influences on the real catalytic performance. The mechanism influencing H_2_O_2_ production remains relatively unclear. Given the complexity of H_2_O_2_ production, the entire dynamic process involving the use of HCO_3_^−^ electrolytes requires further in‐situ monitoring.
7. Due to the complex composition of intermediates, it is important to develop an in‐situ characterization to track intermediates and active sites for the analysis of dynamic electrocatalytic processes, including FTIR, Raman spectroscopy and X‐ray spectroscopy, these help to reveal the dynamics of catalysts, identify surface intermediates under different reaction conditions, and characteristic changes in the associated active sites.
8. So far, only a few devices have been proposed to produce H_2_O_2_. For integrated electrolysis cell for coupled reactions, to achieve high efficiency of H_2_O_2_ production, a combination of ORR is a strategy to realize double FE and H_2_O_2_ yield. In addition, the combination of 2e‐WOR with carbon reduction (CO_2_RR) and hydrogen evolution reaction (HER) were verified to be interesting. For PEC, the positions of the conduction band (CB) and the valence band (VB) of the electrode material must be precisely designed to meet the requirements of the trans‐reduction and oxidation potentials between them. For large‐scale applications, their surface area, activity, stability and suitability for large‐scale applications and industrial costs need to be considered holistically.
9. Methods need to be developed to stabilize and separate the accumulated H_2_O_2_ from the electrolyte in order to extend its use for different purposes. It is essential to consider how the full anodic synthesis mechanism will vary under flow conditions. H_2_O_2_ will be carried away from the electrode surface after the initial two‐electron transfer before the occurrence of side effects. To stabilize hydrogen peroxide, complexing agents, also known as stabilizers, such as sodium silicate and Ethylene Diamine Tetraacetic Acid (EDTA), can be added. These agents form complexes with H_2_O_2_ and reduce its decomposition rate.
10. The electrochemical reactor requires further innovation to improve energy efficiency, facilitate production purification and integrate in‐situ applications, making the production of hydrogen peroxide via 2e‐WOR more promising for industrial applications. Future investigations should also consider the fate of H_2_O_2_ after its electrochemical production. Research of methodologies in this area will be important in moving 2e‐WOR towards device level implementation.
11. The cost of distributed production/application of H_2_O_2_ must be competitive with centralized production and distributed application. Considering the feasibility of industrial application, we need to conduct a techno‐economic analysis and a production cycle analysis to assess the practicality and feasibility of 2e‐WOR hydrogen peroxide production.

These advances are also expected to stimulate the production of a wide range of high‐value chemicals, promoting efficient energy use and environmentally friendly development.

## Conflict of Interests

The authors declare no conflict of interest.

## Biographical Information

*Huixuan Cao is presently enrolled as a PhD student at Beijing University of Technology, where she is being mentored by Professor Ge Chen. Professor Chen′s research group is dedicated to the development of novel electrocatalysts specifically designed for energy conversion*.

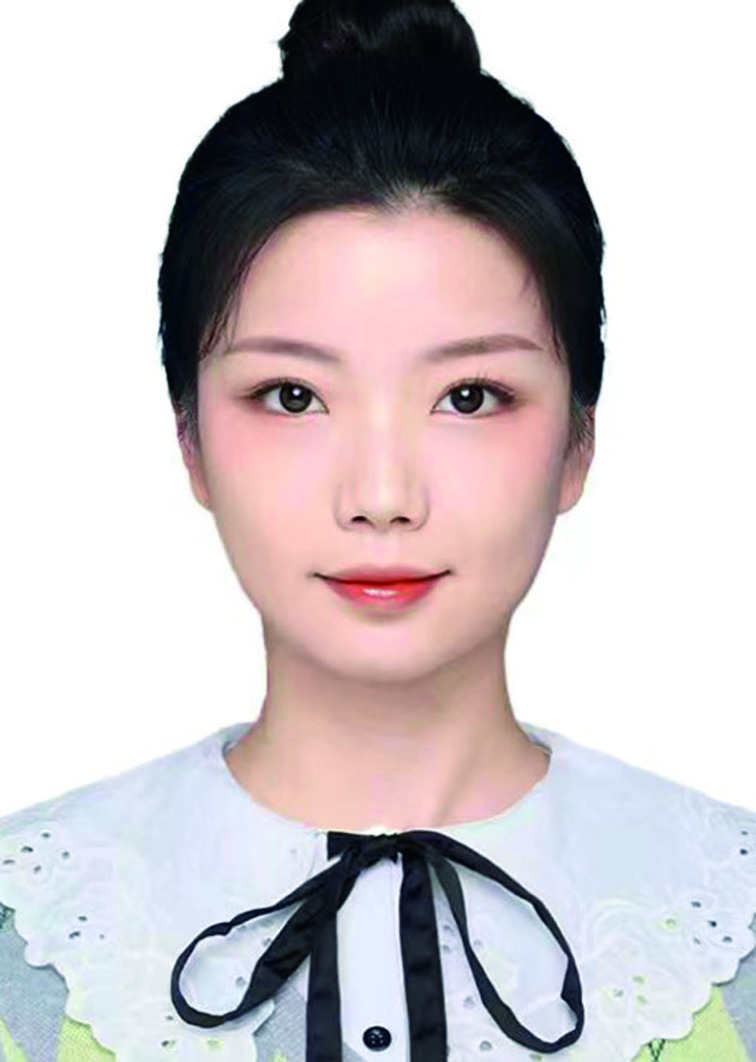

## Biographical Information

*Ge Chen received his BS and MS degrees from the University of Science & Technology, Beijing, in 1997 and 2000, respectively, and PhD degree from the Beijing University of Technology in 2010. He is now a full professor in the Faculty of Environment and Life, Beijing University of Technology. His research group interests are focused on developing novel electrocatalysts towards energy conversion*.

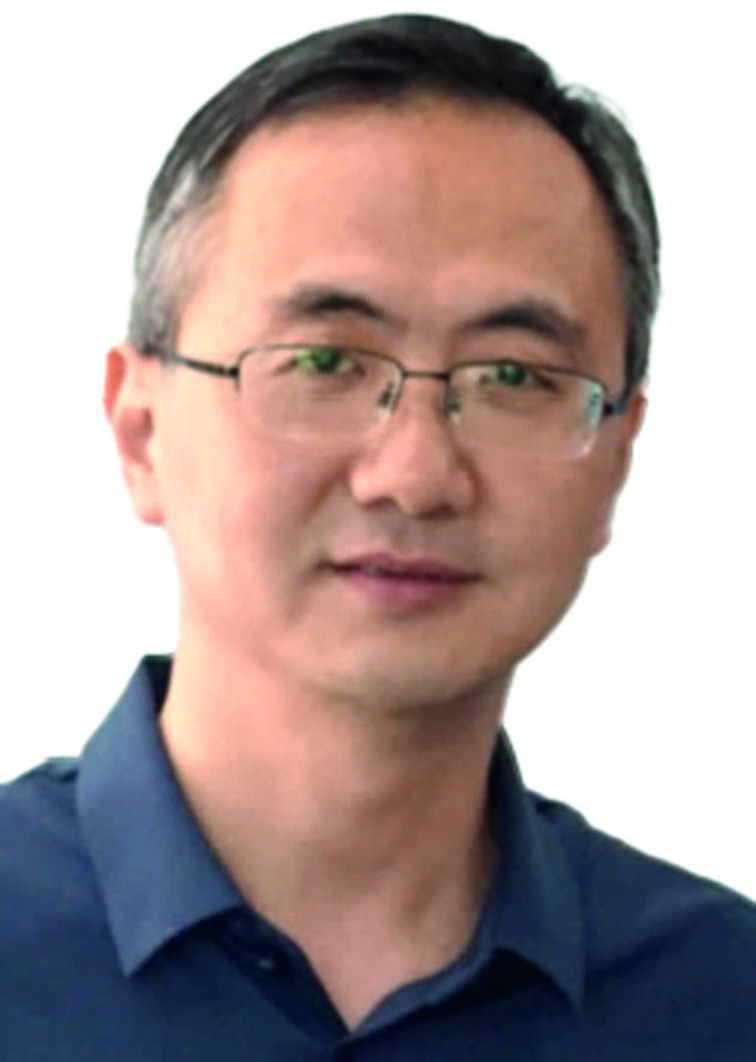

## Biographical Information

*Yong Yan received his Ph.D. degree in 2015 from Ilmenau University of Technology (TU Ilmenau) in Germany. He is an associate professor working at Beijing University of Technology (BJUT) in China. His research mainly focused on electrochemical energy catalysis, electrochemical in situ Raman and infrared spectroscopy, Catalytic oxidation of C‐H bonds at plasma resonance interfaces*.

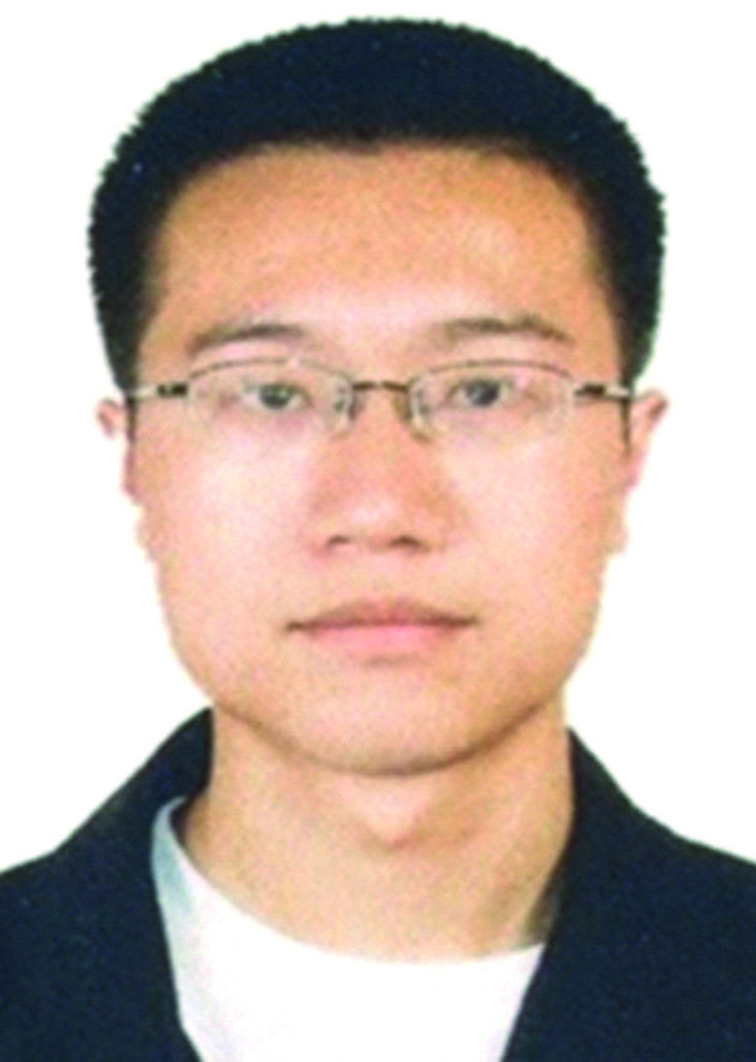

## Biographical Information

*Dong Wang received his Ph.D. degree in 2007 at Karlsruhe University and finished his Habilitation in 2016 at TU Ilmenau. He is now working as an associate professor (Privatdozent) at TU Ilmenau with research focus on tailored functional nanomaterials*.